# Prenatal and childhood exposures to heavy metals and their associations with child cognition, motor skills, behaviour and mental health

**DOI:** 10.1042/EBC20253010

**Published:** 2025-12-29

**Authors:** Deborah Dewey, Munawar Hussain Soomro

**Affiliations:** 1 Department of Pediatrics, Cumming School of Medicine, University of Calgary, Calgary, Canada; 2 Owerko Centre, Alberta Children’s Hospital Research Institute, University of Calgary, Calgary, Canada; 3 Department of Community Health Sciences, Cumming School of Medicine, University of Calgary, Calgary, Alberta, Canada; 4 Hotchkiss Brain Institute, University of Calgary, Calgary, Alberta, Canada

**Keywords:** behaviour, childhood exposure, heavy metals, mental health, prenatal exposure, motor skills, cognition, motor skills

## Abstract

Exposure to arsenic (As), cadmium (Cd), lead (Pb) and mercury (Hg), prenatally and in childhood could pose a significant risk to children’s neurodevelopmental outcomes. A mini-review synthesized the findings of original peer-reviewed prospective cohort studies that investigated associations between prenatal and/or childhood exposure to As, Cd, Pb and Hg and chemical mixtures that included these metals, and cognitive, motor, behaviour and mental health outcomes in children and adolescents. Scopus, OVID Medline, EMBASE and PsychINFO were searched for relevant studies published in English between January 01, 2022, and June 30, 2025. Of the 1089 studies identified, 77 met the criteria for inclusion. Thirty-four different cohorts for 18 countries were included, and sample sizes ranged from 48 to 96,165. Exposure was primarily assessed using biological samples such as maternal and child blood, cord blood, and maternal and child urine. The findings of this review provide strong support for the contention that higher levels of prenatal and childhood exposure to As, Cd, Pb and Hg, and their mixtures are linked with adverse cognitive, motor, behavioural and mental health outcomes in children. There is some suggestion that these effects may differ by child sex. Prenatal and childhood exposure to these toxic metals has lasting consequences for children’s neurodevelopment. Future research that examines the effects of prenatal, early childhood and continued exposure to these toxic metals on adult neurodevelopment is critical. Further, the potential mitigating effects of maternal and child nutrition and the influences of the psychosocial environment on long term outcomes are areas in need of future study.

## Introduction

Heavy metals such as arsenic (As), cadmium (Cd), lead (Pb) and mercury (Hg) are metal elements with densities *>* 5 g/cm3 [1,2]. These metals are ubiquitous environmental pollutants and come from a variety of sources, including agricultural runoff, industrial discharges and air pollution. Primary routes of environmental exposure are through inhalation of indoor and outdoor air pollutants or ingestion of contaminated food and water [1,2]. The Agency for Toxic Substances and Disease Registry has placed As, Cd, Pb, and Hg and its compounds including methylmercury (MeHg) on the Priority Substance List, which includes substances that have been determined to pose a most significant potential threat to human health [3]. Exposure to these metals has been associated with cellular damage, oxidative stress, and inflammation, and disruption in cell growth, neural development and hormonal regulation [4–10].

Exposure to As, Cd, Pb, Hg and MeHg during foetal development and childhood could have serious and long-lasting consequences for children’s cognitive, motor and behavioural development and mental health [9,11–14]. These metals can cross the placental barrier and directly affect the foetal brain [9,15–17]. Children are more vulnerable to toxic metal exposure than adults because their higher metabolic and physiologiucal activity results in greater absorption of metals relative to body weight [9,18–20]. Also, children’s behaviours, including putting objects in their mouths and playing on the ground, increase their risk of exposure to metals found in dust and soil [21]. Further, the detoxification systems of children are immature, limiting their ability to effectively process and eliminate harmful substances [22]. Thus, prolonged exposure to toxic metals, even low doses across childhood and into adolescence, heightens the risk of adverse cognitive, motor, behavioural and mental health outcomes that may persist throughout childhood and into adulthood [23–25].

Much of the previous research that has examined the associations between exposure to heavy metals and child neurodevelopment has focused on the impact of individual metals. However, as co-exposure to multiple metals and other chemical elements is typical, recent research has examined the joint effects of exposure to chemical mixtures. The primary aim of this mini-review was to synthesize the findings of prospective cohort studies published within the last three years that investigated the associations between prenatal and/or childhood exposures to As, Cd, Pb, Hg and MeHg and metal mixtures that included these metals, and children’s cognition, motor, behavioural and mental health outcomes.

## Methods

This mini-review followed the guidelines outlined in the Preferred Reporting Items for Systematic Reviews and Meta-Analyses (PRISMA). A literature search was conducted on June 30, 2025. A systematic search of OVID Medline, EMBASE, Scopus and PsychINFO, restricted to original peer-reviewed prospective cohort studies published in English between January 1, 2022, and June 30, 2025, was conducted. Table 1 includes a detailed description of the inclusion and exclusion criteria.

**Table 1:** Inclusion and exculsion criteria for studies included in this mini-review

Include	Exclude
1. Peer-reviewed prospective cohort studies.	1. Observational case-control studies, case reports, cross-sectional studies, systematic reviews, scoping reviews, narrative reviews.
2. Investigated exposure to As, Cd, Pb and/or Hg or MeHg and metal mixtures.	2. Did not include at least one of As, Cd, Pb and/or Hg or MeHg.
3. Measures of As, Cd, Pb and/or Hg or MeHg in pregnant women and/or children 0–18 years.	3. Measures of As, Cd, Pb and/or Hg or MeHg in other populations.
4. Direct measures of As, Cd, Pb and/or Hg or MeHg concentrations in biological sample (i.e. blood, cord blood, urine, placenta, hair, human milk, meconium).	4. Indirect measures of As, Cd, Pb and/or Hg or MeHg from food consumption, exposure to drinking water or air pollution.
5. Standardized measures of cognition, motor skills, behaviour or mental health in children.	5. Experimental measures of cogniton, motor skills, behaviour or mental health in children.

As, Arsenic. Cd, Cadmium. Pb, Lead. Hg, Mercury. MeHg, Methylmercury.

The following search terms were used: ((arsenic OR cadmium OR lead OR mercury OR heavy metals) AND (cognition OR intelligence quotient OR neurodevelopment OR neurodevelopmental outcome OR neurodevelopmental disorders OR behavior OR behavior disorders OR externalizing behavior OR internalizing behavior OR ADHD OR attention deficit hyperactivity disorder OR autism OR ASD OR autism spectrum disorder OR mental health OR anxiety OR depression) AND (prenatal exposure OR maternal exposure OR childhood exposure) AND (infant OR children OR adolescent)). Relevant data from the studies included in this review were extracted, including the first author’s name, publication year, cohort, country where the study was conducted, participant sample size, metal/chemical exposures, time exposure measured, outcomes, child age at outcome assessment, main findings and biological samples used to measure exposure.

## Results

### Study selection

**Figure 1:**
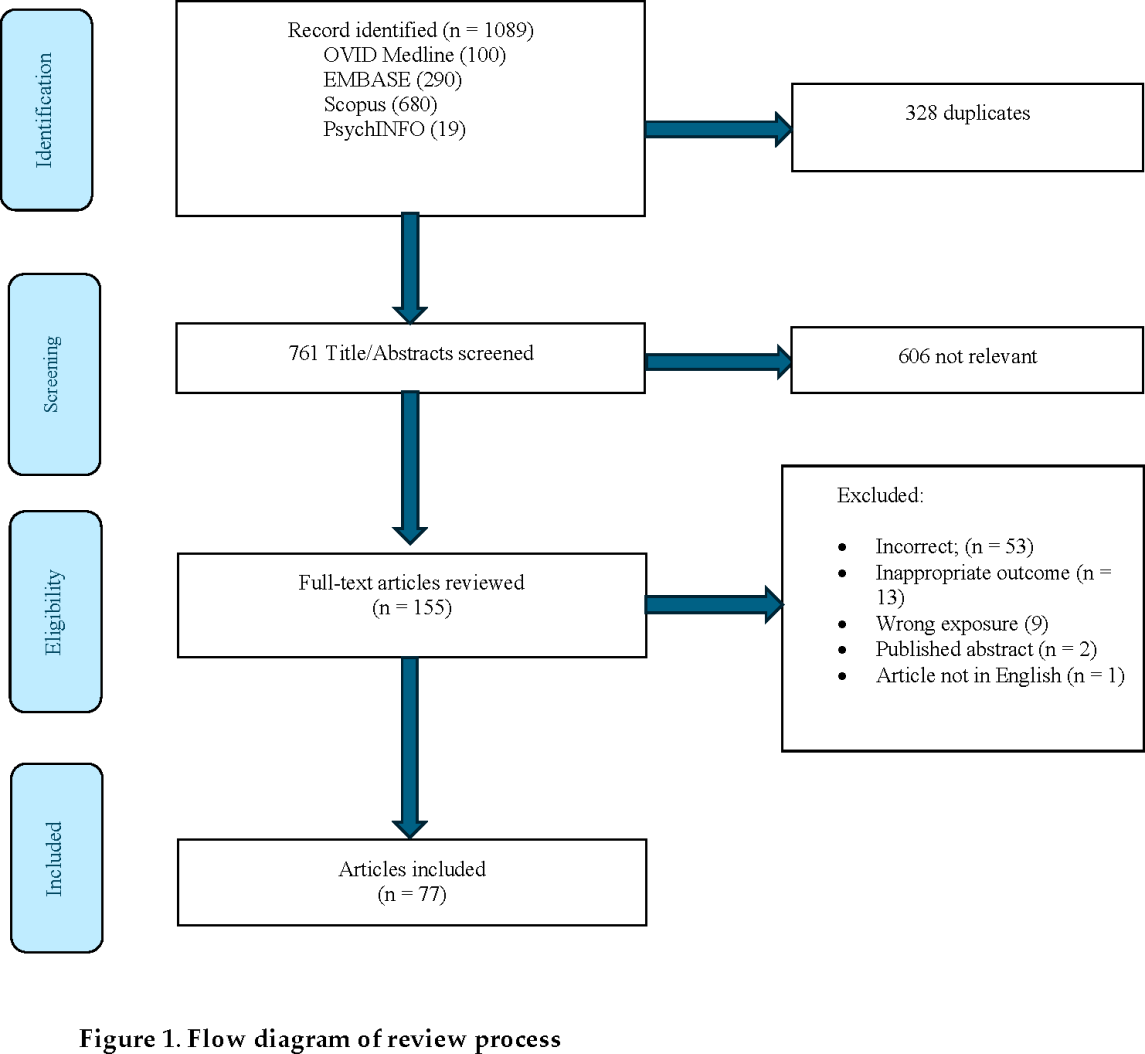
Flow diagram of review process.

Our initial search identified 1089 articles. After removal of duplicates, 761 studies underwent title and abstract screening and 155 full texts were reviewed; of these, 77 articles were identified for inclusion (see Figure 1). Thirty-five studies examined the influence of exposure to either As, Cd, Pb, Hg, or MeHg) on children’s cognitive, motor, behavioural and/or mental health outcomes; 8 focused on As, one examined the influence of Cd, 16 investigated the influence of Pb and 10 examined Hg and/or MeHg. Eighteen studies investigated associations between multiple toxic metals and/or other chemical exposures (i.e. phthalates, polychlorinated biphenyls, fluoride, per- and polyfluoroalkyl substances (PFAAs), essential metals) and child outcomes. Twenty-four studies investigated the joint influence of metal mixtures on child outcomes.

### Study descriptives

Research examining exposure to As, Cd, Pb, Hg and MeHg (and other metal and chemicals) was conducted in 34 cohorts from different countries/regions including China (*n* = 20), USA (*n* = 17), Canada (*n* = 8), Mexico (*n* = 6), Japan (*n* = 6), Spain (*n* = 5), Taiwan (*n* = 3), Seychelles (*n* = 3), Brazil (*n* = 2), Guadeloupe (*n* = 1), Sweden (*n* = 1), Norway (*n* = 1), Poland (*n* = 1), Suriname (*n* = 1), Italy (*n* = 1), Tanzania (*n* = 1), and Palestine (*n* = 1). The majority of studies examined heavy metals levels in blood, cord blood and/or urine; however, concentrations in other biomarkers were also investigated including hair, placental tissue, meconium, human milk, human bone and nails [26–37]. Most studies investigated associations between prenatal exposure and child outcomes (65%, *n* = 50); however, some studies investigated childhood exposure only (17%, *n* = 13), while others examined both prenatal and childhood exposures (17%, *n* = 13). Cohort sample sizes ranged from 48 to 96,165. Standardized measures used to assess children’s neurodevelopment outcomes included direct assessments of child performance (e.g. Bayley Scales of Infant Development (BSID), Wechsler Preschool and Primary Scales of Intelligence (WPPSI)) and parent reports of child development, behaviour and mental health (e.g. Ages and Stages Questionnaire (ASQ) Child Behavior Checklist (CBCL), Behavior Assessment System for Children (BASC), Strengths and Difficulties Questionnaire (SDQ), Connor Parent Rating Scale (CPRS)).

### Arsenic exposure and child cognition, motor skills, behaviour and mental health

#### Cognition/general development

In both single chemical exposure studies (see Table 2) and studies that examined multiple chemicals (see Table 3), higher levels of maternal urinary As during pregnancy were associated with poorer cognitive/developmental outcomes in childhood [51–54,74]. Chen reported that higher levels of inorganic As and total As were associated with lower scores on the Mental Development Index (MDI) of the BSID at two years of age [51] . Similarly, Soler-Basco found that higher levels of monomethyl arsenic acid (MMA) were associated with lower overall cognitive ability on the McCarthy Scales of Children’s Abilities (MSCA) at four to five years of age [54]. However, associations between As exposure and cognitive/developmental outcomes may vary with child age at assessment. In a longitudinal follow-up study, Signes-Pastor et al. found that maternal As was not associated with children’s mental development at one, two and three years [53]. At five years, higher levels of maternal As were associated with lower Full Scale IQ (FSIQ; intelligence); however, by eight years this association was no longer significant. Fewer studies have investigated associations between prenatal exposure to As in other biosamples (i.e. maternal blood, cord serum); however, Li et al. noted that As levels in cord serum were inversely associated with IQ scores on the Fluid Reasoning and Working Memory Indices of the WPPSI-IV in children 2.5–6 years of age [45]. They also noted that some of these associations were sex-specific.

**Table 2:** Single chemical studies that examined associations between prenatal or childhood exposure to arsenic, cadmium, lead or mercury in maternal biosamples and cognitive, motor, behavioural and mental health outcomes in children

Study/ Cohort	Country	Sample size	Exposure	Time exposure measured	Outcome	Child age at outcome assessment	Main findings
*Maternal blood*							
Masumoto et al. 2022/ Japan Environment and Children's Study [38]	Japan	96,165 mother– child pairs	Cd	2nd or 3rd trimester	Ages and Stages Questionnaire (ASQ) -3 -Japan Communication Gross Motor Function Fine Motor Function Problem Solving Personal–Social delay classified as a score > 2 SD below the mean	6 months, 1 years, 1.5 years, 2 years, 2.5 years and 3 years after birth	↑ Cd ↑ odds of delay in overall child development (i.e. a score on one domain of three > 2 SD below the mean) at 6 months (aOR = 1.10, 99.7% CI 1.01–1.20), 1 year (aOR = 1 year 1.13, 99.7% CI 1.02–1.24), and 1.5 years (aOR = 1.15, 99.7% CI 1.03–1.28) At 1 year ↑ Cd ↑ odds of delay in Gross Motor Function (aOR = 1.25, 99.7% CI 1.07–1.45) and Problem-Solving Skills (aOR = 1.22, 99.7% CI 1.05–1.42) At 1.5 years ↑ Cd ↑ odds of delay in gross motor function (aOR = 1.19, 99.7% CI 1.01–1.40)
Alampi et al. 2024/ Maternal -Infant Research on Environmental Chemicals (MIREC) [39]	Canada	601 mother–child pairs	Pb	1st and 3rd trimesters	Social Responsiveness Scale (SRS)-2	3–4 years	↑ 1st trimester Pb ↑ SRS-2 (*β* = 0.4; 95% CI: −0.3, 1.1) ↑ 3rd trimester Pb ↑ SRS-2 in participants with low ( < 10 th percentile) 3rd-trimester plasma total folate (*β* = 3.3; 95% CI: 1.1, 5.5)
Jia et al. 2023/ Healthy Baby Cohort [40]	China	2361 mother–child pairs	Pb	1st trimester	Bayley Scales of Infant Development (BSID)-China Mental Development Index (MDI) (score of less than or equal to 79 indicative of Cognitive Developmental Delay (CDD))	2 years	↑ Pb ↑ odds of cognitive developmental delay (CDD) (highest vs. lowest tertile: adjusted OR, 1.55; 95% CI:1.13–2.13); similar findings when adjusted for child Pb. ↑ Pb ↑ CDD was stronger in girls (highest vs. lowest tertile; OR, 2.04; 95% CI:1.16–3.58) ↑ Pb higher odds of ↑ CDD in children with polygenetic risk scores (highest vs. lowest tertile OR, 2.59; 95% CI: 1.48–4.55)
Kadawagedara et al. 2023/ ZIKA-DFA-FE study [41]	Guadeloupe	297 mother–child pairs	Pb	At delivery	ASQ-French Total Fine motor Problem Solving Quebec Longitudinal Study of Child Development: Externalizing Behaviours Hyperactivity Opposition Inattention	18 months	↑ Pb ↓ ASQ Total (β = − 19.3; 95% CI: − 39.8; 1.4); ↑ Pb ↓ ASQ Fine Motor (β = − 5.9; 95% CI: − 12.2; 0.5) ↑ Pb ↓ ASQ Problem Solving (β = − 14.6; 95% CI: − 21.7; − 7.6); vulnerability noted in boys.
Koendjbiharie et al. 2023/ Caribbean Consortium for Research in Environmental and Occupational Health (CCREOH) MeKi Tamara Study [42]	Suriname	363 mother–child pairs	Pb	1st or early 2nd trimester	BSID-III Cognitive Language (Receptive, Expressive) Motor (Fine, Gross)	12–24 months	Pb ≥ 3.5 µg/dL ↓ Receptive Language (β −0.26, 95% CI: −0.49,–0.02)
Packull-McCormick et al. 2023/ MIREC [43]	Canada	527 mother–child pairs	Hg	1st trimester	Wechler Preschool and Primary Scale of Intelligence (WPPSI)-III Full Scale IQ (FSIQ) Verbal IQ (VIQ) Performance IQ (PIQ)	3–4 years	No significant associations
Vejrup et al. 2022/ Norwegian Mother, Father and Child Cohort Study (MoBa)[44]	Norway	-2936 mother– child pairs	MeHg	18 weeks gestation	Child Behaviour Checklist (CBCL): 26	3 and 5 years	No significant associations
*Cord blood*							
Li et al. 2025/ Ma'anshan Birth Cohort (MABC) [45]	China	1814 mother–child pairs	As	At birth	WPPSI–IV-China FSIQ Verbal Comprehension Visual Spatial Fluid Reasoning Working Memory Processing Speed	2.5 to 6.0 years	↑ As levels ↓ Fluid Reasoning index IQ (β = − 1.07; 95% CI: − 1.98,–0.16) and ↓ Working Memory index (WMI) IQ (β = − 1.51; 95% CI: − 2.76,–0.25). ↑ As ↓ WMI IQ in males (β = − 1.51; 95% CI: − 2.76,–0.25).
Lu et al. 2023/ Prospective Cohort, Shanghai [46]	China	275 mother–child pairs	Pb	At birth	BSID-III-China Cognitive Language (Receptive, Expressive) Motor (Fine, Gross)	18 months	↑ Pb ↓ Fine Motor scores in female children (β = −1.5; 95% CI: −2.6,–0.4
Kuraoka et al. 2024/ Japan Environment and Children’s Study [47]	Japan	3083 mother– child pairs	MeHg; inorganic Hg (iHg)	At birth	Kyoto Scale of Psychological Development; Developmental (quotient score of < 70 indicated negative outcome)	2 and 4 years	No significant associations
Love et al. 2022/ (SCDS NC 2) [48]	Seychelles	973 mother–child pairs	Total Hg (THg) was presumed to be primarily MeHg	At birth	BSID-II MDI Psychomotor Development Index (PDI)	20 months of age	↑ MeHg ↓ MDI (slope=−0.091, *P* = 0.014) in children homozygous for the rare C-allele in ABCB1 rs10276499
Sarzo et al. 2024/ INfancia y Medio Ambiente (INMA) cohort [49]	Spain	1147 mother–child pairs	THg	At birth	ADHD-DSM-IV diagnosis Strength and Difficulties Questionnaire Connor Parent Rating Scale (CPRS) CBCL	4, 7, 9 and 11 years,	No significant associations
*Maternal hair*							
Thurston et al. 2025/ New Bedford Cohot [29]	USA	361 mother–child pairs	MeHg	10 days postpartum	Wechsler Intelligence Scale for Children (WISC)-III Boston Naming Test (BNT) Wide Range Assessment of Memory and Learning (WRAML)	8 years	No significant associations
*Maternal urine*							
Butler et al. 2023/ New Hampshire Birth Cohort (NHBCS [50]	USA	395 mother–child pairs	Total As (TAs)	24–28 weeks GA	Bruininks-Oseretsky Test of Motor Proficiency- 2nd Edition (BOT-2): Short Form and fine motor items from Complete BOT-2	5.5 years	TAs > 9.5 ug/L ↓ motor proficiency (ß = -3.3; 95% CI: −6.1.–0.4) TAs > 17.0 ug/L ↓ fine motor by 4.3 point (ß = -4.3: 95% CI: −8.0,–0.6)
Chen et al. 2023/ Wuhan Medical & Healthcare Center for Women and Children [51]	China	1006 mother– child pairs	As species: arsenobetaine (AsB) arsenite arsenate monomethyl arsenic acid (MMA) dimethyl arsenic acid (DMA) inorganic arsenic (iAs) was calculated as the sum of arsenite and arsenate total arsenic (tAs) was calculated as the sum of iAs, MMA and DMA	1st, 2nd and 3rd trimesters	BSID-China MDI PDI	2 years	↑ iAs ↓ MDI (ß = - 2.45; 95% CI: −4.86,–0.05) ↑ tAs ↓ MDI (ß = -2.57; 95% CI: −5.22, 0.07) Trimester effects: ↑ As and ↓ MDI in 1st and 2nd trimester ↑ As and ↓ PDI in 1st trimester
Patti et al. 2022/ MIREC [52]	Canada	364 mother–child pairs	As species: arsenite arsenate DMA MMA AsB	1st trimester	WPPSI-III SRS-2 Behaviour Assessment System for Children (BASC) Behavioral Symptoms Index Internalizing Problems Externalizing Problems Adaptive Skills School Problems optimal neurodevelopmental profile charctereized by higher than average FSIQ and lower than average SRS and BASC scores; typical neurodevelopment characterized by average FSIQ and lower than average SRS and BASC scores	3 years	↑ DMA ↓ likelihood of the optimal neurodevelopmental phenotypic profile compared with the typical neurodevelopmental phenotypic profile (OR: 0.44; 95% CI: 0.19, 1.02)
Signes-Pastor et al. 2022/ HOME [53]	USA	260 mother–child pairs	As species: DMA MMA AsB arsenite arsenate ∑As was the sum of arsenate, MMA, and DMA.	16- and 26 week gestation	BSID-II WPPSI-III WISC-IV	1, 2, 3, 5 and 8	∑As not significantly associated with MDI at 1, 2 or 3 years. At 5 years, ↑ ∑As ↓ Full Scale IQ (FSIQ) (*β* = 2.5; 95% CI; -5.1, 0.0). At 8 years, ∑As not significantly associated with FSIQ
Soler-Blasco et al. 2022/ INMA [54]	Spain	807 mother–child pairs	TAs As species: DMA MMA iAs AsB	1st trimester	McCarthy Scales of Children’s Abilities (MSCA) General Scale Verbal Scale Quantitivative Scale Memory Scale Executive Function Scale Working Memory Scale	4–5 years	↑ MMA ↓ MSCA General Scale (*β* = 1.37; 95% CI; −2.33,–0.41), Verbal Scale (*β* = 1.18 95% CI: −2.13,–0.23), Quantitative Scale (*β* = 1.23; 95% CI: −2.20,–0.27), Memory Scale (*β* = 1.19; 95% CI: −2.17,–0.20), Executive Function Scale (*β* = 0.98; 95% CI: −2.00, 0.04, and Working Memory Scale (*β* = 0.96; 95% CI: −1.90,–0.02. ↑ TAs and ↑ Asb ↑ Verbal Scale (*β* = 0.65; 95% CI:0.03, 1.27, *β* = 0.59; 95% CI: 0.11, 1.07, respectively)
Wylie et al. 2025/ Brain and Early Experience (BEE) [55]	USA	107 mother–child pairs	Pb	25–38 weeks gestational age	MacArthur-Bates Communicative Development Inventory-III Early Childhood Behavior Questionnaire Effortful Control scales EF touch: computerized battery of executive function skills StimQ-Infant; cognitive home environment	28–39 months and 36–49 months	No associations with language, effortful control or executive function outcomes When cognitive home environment scores were low, ↑ Pb ↓ child language (*β* = −0.32, *P*=0.04).
*Maternal blood and cord blood*							
Guo et al. 2022/ Shanghai Birth Cohort [56]	China	2132 mother– child pairs	Pb	< 16 weeks’ GA; at birth	ASQ-3-China ASQ: Social Emotional (ASQ:SE) BSID-III-China Cognitive Motor Language Socio-Emotional Adaptive Behavior	ASQ-3 and ASQ:SE: 6 to 12 months BSID-III:24 months	Maternal blood: At 24 months: Pb + Stress group (maternal blood Pb ≥ 2 μg/dL and maternal depression or/and anxiety scores ≥ 70 percentile) ↓ social emotional development on BSID-III compared with Controls (Pb < 2 μg/dL and maternal depression or/and anxiety scores < 70 percentile) (*β* = 10.48, 95 % CI: −17.42,–3.54) Pb group (maternal blood Pb ≥ 2 μg/dL and maternal depression or/and anxiety scores < 70 percentile) ↑ motor development on BSID-III than Controls (*β* = 5.80, 95 % CI: 0.37, 11.23) Cord blood: At 6 months: Pb + Stress group ↓ ASQ-3 Communication scores than Controls (*β* = −3.79, 95 % CI: 6.32,–1.26) At 24 months, Pb + Stress group BSID-III Social-Emotional development than Controls (*β* = −5.95, 95 % CI: −11.53,–0.36)
Inoue et al. 2022/ Japan Environment and Children’s Study [57]	Japan	80,759	Pb	2nd/3rd trimesters; at birth	ASQ-3-Japan suspected neurodevelopmental delay (sNDD) classified as a score > 2 SD below the mean	Children assessed at 6, 12, 18, 24, 30, and 36 months	↑ maternal Pb ↓ risk of sNDD at 3 years (adjusted RR = 0.84, 95% CI: 0.75–0.94) Cord blood: no associations
*Child blood*							
Dai et al. 2024/ China Jintan Child Cohort [58]	China	972 adolescents	Pb	3–5 and 11–13 years	Child Sleep Health Questionnaire–parent report Pittsburgh Sleep Quality Index – adolescent self-report	11–13 years	↑ Pb at 3–5 years ↑ parent-reported sleep onset delay, and ↑ night waking ↑ Pb 11–13 years, ↑ short sleep duration, ↑ night wakings ↑ parasomnias and ↑ sleep disordered breathing Most associations significant in males In female adolescents ↑ Pb levels ↓ self-reported sleep duration.
Halabicky et al. 2022/ Jintan Child Cohort [59]	China	681 children	Pb	3–5 years and 12 years	University of Pennsylvania Computerized Neurocognitive Battery: Abstraction /Flexibility Attention Spatial Processing Episodic Memory Sensorimotor Speed Motor Speed	12 years	↑ Pb at 3–5 ↑ time to correctly complete Abstraction/Flexibility tasks (*β* = 19.90, 95% CI, 4.26, 35.54) and Spatial Processing tasks (*β* = 96.00, 95% CI, 6.18, 185.82) in males ↑ Pb at 3–5 ↑ time to correctly complete Episodic Memory Tasks (*β* = 34.59, 95% CI 5.33, 63.84) in females In males, ↑ Pb 12 years ↑ time for correct responses on the Attention Tasks (*β* = 15.08, 95% CI, 0.65, 29.51) and ↓ time for correct responses on the Episodic Memory tasks (*β* = -73.49, 95% CI −138.91,–8.06)
Halabicky et al. (2023)/ Jintan Child Cohort [60]	China	417 children	Pb	3–5 years and 12 years	WISC-Revised-China FSIQ Working Memory Measurement Software: visuospatial numeric	12 years	↑ Pb at 3–5 years ↓ FSIQ at 12 years (*β* = -0.55 95% CI: −0.97,–0.13) ↑ Pb concentrations at 3–5 and 12 years ↓ FSIQ (β = −3.91, 95% CI; −6.11,–0.27) and Working Memory (β = −1.24, 95% CI; −2.24,–0.06) compared with children with persistently low or low/high or high/low Pb concentrations ↑ Pb concentrations and ↓ paternal education had FSIQ scores 7.84 (95% CI; −13.15,–2.53) points lower than children with ↓ Pb concentrations and ↓ paternal education.
Min et al. 2022/ Midwest United States [61]	USA	265 low SES, urban, primarily African American youth	Pb	4 years	WISC-IV Test of Language Development-Intermediate-3 CBCL The Youth Risk Behavior Surveillance System	11, 12 and 15 years	↑ Pb ↓ language skills at 12 years (β = −0.149, 95% CI: −0.269,–0.029) ↑ Pb ↑ greater likelihood of substance use at 15 years (*β* = 0.16; 95% CI: 0.004,–0.327) Language skills mediated association between Pb and substance use-related problems (e.g. drunk driving, feelings of addiction, missed school) at 15 years ((β = −0.364; 95% CI: −0.522,–0.205)
Sears et al. 2022/ Health Outcomes and Measures of the Environment (HOME) Study [62]	USA	244 children	Pb	1,2,3,4,5 and 8 years	BASC-2 Behavioral Symptoms Index Internalizing Problems Externalixing Problems Adaptive Skills School Problems	2,3,4,5 and 8 years	8 years, ↑ Pb ↑ Externalizing Problems (*β* = 3.1; 95% CI: 0.7, 5.4) ↑ Pb ↓ Adaptive Skills (β=−2.2; 95% CI: −4.9, 0.5)
*Child hair*							
Thurston et al. 2022 (SCDS NC 2) [28]	Seychelles	312–550 children/adults	MeHg	Time weighted average exposure for childhood (TW-C) = 6 months to 5.5 years; time weighted average exposure for early adulthood (TW- A) = 17 to 24 years	85 neurodevelopmental outcomes: executive function attention cognition achievement verbal learning and memory fine motor speed	9, 10.5, 17, 19, 22, and 24 years	↑ TW-C Hg ↑ Continuous Performance Test risk score (slope = 0.753; CI = 0.032, 1.474) at 9 years At 22 years, ↑ TW-C Hg ↓ BNT total (slope = -0.253; CI = -0.504,–002) and not cue score (slope = -0.283; CI = -0.523,–0.043) At age 24 years, ↑ TW-C Hg ↓ auditory response time (slope = 0.014; CI = 0, 0.02), visual response time (slope = 0.006, CI = 0, 0.012) and visual response time variance (slope = 0.015; CI = 0.003, 0.026). ↑ TW-A Hg at 17 years, ↑ Wisconsin Card Sorting Test % error (slope = 0.533; CI = 0.005, 1.061), ↑ CANTAB Rapid Visual Information Processing false alarms (slope = 0.017; CI = 0.002, 0.031) and ↓ Woodcock Johnson Passage Comprehension (slope = -0.419; CI = -0.801,–0.037) At age 22 years, ↑ TW-A ↑ Intra-Extra Dimensional Shift total errors (slope = 0.022; CI = 0.005, 0.04) and total trials (slope = 0.011; CI = 0.003, 0.019), and ↓ BNT total score (slope = -0.222; CI = -0.39,–0.054) and ↓ BNT no cues (slope = -0.222; CI = -0.383,–0.061)
Nakamura et al. 2023/ First graders in Kinan region, Wakayama Perfecture [63]	Japan	151 children	MeHg	7–8 years	WISC-III BNT	7–8 years	No significant associations
*Cord blood and child blood*							
Packull-McCormick et al. /MIREC (2023) [43]	Canada	403 mother–child pairs	Hg	At birth and 3–4 years	WPPSI-III FSIQ VIQ PIQ General Language Composite (GLC)	3–4 years	Cord Blood: ↑ Hg ↑ FSIQ (ß = 1.29, 95% CI = 0.77, 1.81) and PIQ (ß = 2.01, 95% CI = 1.19, 2.83) in female children Child Blood: In female children, ↑ Hg ↑ FSIQ (ß = 0.97, 95% CI = 0.09, 1.84), VIQ (ß = 1.04, 95% CI = 0.41, 1.67), and GLC (ß = 1.09, 95% CI = 0.23, 1.82)
Liu Child et al. 2024/ Programming Research in Obesity, Growth, Environment and Social Stressors (PROGRESS) cohort [64]	Mexico	533 mother–child pairs	Pb	At birth and 4 years	Inhibitory control tasks: Go/NoGo Letter Go/NoGo Neutral Go/NoGo Happy Delis-Kaplan Color-Word Interference Test	8–9 years	Cord Blood: ↑ Pb ↓ inhibitory control at 8–10 years (*β* = --0.06, 95% CI: −0.10,–0.02) ↑ Pb ↓ inhibitory control in males (β = − 0.09, 95% CI: −0.14,–0.04) at 8–9 years Child Blood: ↑ Pb ↓ inhibitory control in males (*β* = -0.05, 95% CI: −0.10,–0.01) and females (*β* = −0.09 (95% CI: −0.14,–0.03) at 8–9 years
*Cord blood and child hair*							
Nakamura et al. 2023/ First graders in Kinan region, Wakayama Perfecture [63]	Japan	151 mother–child pairs	MeHg	Cord blood: at birth child hair: 7–8 years	WISC-III BNT	7–8 years	No significant associations
*Maternal bone, cord blood, child blood*							
Reyes Sanchez 2022/ELEMENT [27]	Mexico	743 maternal exposure and cord blood 704 early childhood exposure 595 periadolescent exposure	Pb	Maternal patella measured at 1 and 12 months postpartum, cord blood; child blood: at birth and 60 months (early exposure) and periadolecence (6–18 years)	BASC-2 Conduct Problems subscale Aggression subscale	6–11 years, 12–18 years	Maternal Bone: ↑ Pb ↑ Conduct Problems at 6–11 years; (OR = 1.31, 95 % CI: 1.01, 1.70). ↑ Pb and ↑ Aggression at 6–11 years (OR = 1.24, 95 % CI: 0.93, 1.65). . Child blood at birth, 60 months or 6–18 years: no significant associations
*Maternal and child hair*							
Klus et al. 2023/ Seychelles Child Development Study: Nutrition Cohort [65]	Seychelles	376 mother–child pairs	MeHg	At delivery	Clinical Evaluation of Language Fundamentals- 5 Kaufman Brief Intelligence Test (KBIT)-2 BNT Trail Making A Finger Tapping Woodcock-Johnson Test of Achievement–3 CBCL SRS- 2 Social Communication Questionnaire	7 years	Maternal hair: no significant assocations Child Hair: no significant associations
*Maternal and child urine*							
Dai et al. 2023/ Sheyangmini Birth Cohort Study (SMBC)[66]	China	389 mother–child pairs	As	During pregnancy and at 6 years	CBCL Anxious/depressed subscle	6 years	Maternal Urine: no significant associations Child Urine: ↑ As ↑ Anxious/Depressed Problems in girls (*β* = 0.71, 95% CI: 0.12–1.31)
*Child hair and urine*							
Ehlinger et al. 2025/ELEMENT [26]	Mexico	399 children	Hg	6–12 years	Connors Continuous Performance Test, 3^rd^ Edition Inattentivenss Vigilence Impulsivity	M = 14.3 year (SD = 2.1)	Child Hair: ↑ Hg ↓ reduced Inattentiveness (β = −0.02, SE = 0.0095, *P*=.027) ↑ Hg ↓ Vigilance in males (*β* = 1.31, SE = 0.65, *P*=.045) ↑ Hg ↓ Inattentiveness in females (*β* = -0.026, SE = 0.013, *P*=.045) Child urine: ↑ Hg ↓ Impulsivity in females (*β* = -0.034, SE = 0.017, *P*=.048)

Note: aOR = adjusted odds ratio; As = Arsenic; AsB = arsenobetaine; ASQ = Ages and Stages Questionnaire; ASQ:SE = Ages and Stages:Social Emotional; BASC = Behavior Assessment System for Children; BNT = Boston Naming Test; BOT-2 = Bruininks-Oseretsky Test of Motor Proficiency-2nd Edition; BSID = Bayley Scales of Infant and Toddler Development; CBCL = Child Behavior Checklist; Cd = Cadmium; CDD = Cognitive Developmental Delay; CI = Confidence Interval; DMA = Dimethyl Arsenic Acid; FSIQ = Full Scale IQ; Hg = Mercury; iAS = inorganic Arsenic; iHg = inorganic Mercury; MCSA = McCarthy Scales of Children’s Abilities; MMA = Monomethyl Arsenic Acid Hg = Mercury; MDI = Mental Development Index; MeHG = Methylmercury; Pb = Lead; PDI = Psychomotor Development Index; sNDD = suspected Neurdevelopmental Delay; SRS = Social Responsiveness Scale; TAs = Total Arsenic; THg = Total mercury; WISC = Wechsler Intelligence Scale for Chidlren; WPPSI = Wechler Preschool and Primary Scale of Intelligence; WRAML = Wide Range Assessment of Memory and Learning; WRAML = Wide Range Assessment of Memory and Learning

**Table 3:** Studies comprising multiple chemicals including arsenic, cadmium, lead and/or mercury that examined individual chemical associations between prenatal and/or childhood exposure and cognitive, motor, behavioural, and mental health outcomes

Study/ Cohort	Country	Sample size	Exposure	**Time exposure** **measure**	Outcome	Child age at outcome assessment	Main findings
*Maternal blood*							
Farias et al. (2022)/ Mexico’s National Institute of Public Health [67]	Mexico	200 mother-child pairs	Pb, Hg, Mn	3rd trimester	Bayley Scales of Infant Development (BSID)-III Cognitive Language Motor	1, 3, 6, and/or 12 months.	↑ Pb ↓ Language across ages (*β* = −0.15, 95% CI: −0.24–−0.56); association moderated by exposure to Hg and Mn
Midya. (2024)/PROGRESS [68]	Mexico	112 mother– child pairs	Pb, As, Cd, Mn, Co, Zn, Cr, Cs, Cu, Sb, Se	2nd and 3rd trimesters	Child Depression Inventory 2: Self-Report Short version	9–11 years	No significant associations for Cd, or Pb 3rd trimester ↑ As ↓ depression (*β* = -0.03; 95% CI: −0.06,–0.01)
Reardon et al./ Alberta Pregnancy Outcomes and Nutrition (APrON) (2023) [69]	Canada	424 mother–child pairs	Hg, 25 perfluoroalkyl acids (PFAAs)	2nd trimester	BSID-III-Canada Cognitive Language Motor Socio-Emotional Adaptive Behavior	2 years	No significant associations with Hg
*Cord blood*							
Wang et al. (2022) [70]	China	148 mother– children	Pb, Se, As, Cu, Mn, Cr	At birth	Wechsler Intelligence Scale for Children (WISC)-China Full Scale IQ (FSIQ) Verbal IQ (VIQ) Performance (PIQ)	7.5 years	↑ Pb ↓ PIQ (*β* = −0.109; 95% CI: −0.21,–0.007) No significant associations for As
*Cord blood and maternal hair*							
Gari et al. 2022/ Polish Mother and Child Cohort (REPRO_PL) [31].	Poland	436 mother–child pairs	Pb, Cd, Hg, Se, Zn, Cu	3rd trimester maternal hair and cord blood at birth	Intelligence and Development Scale-Poland Fluid IQ Crystallized IQ Mathmatical Skills Strength and Difficulties Questionnaire (SDQ) Total Difficulties Emotional Symptoms Conduct Problems Hyperactivity/Inattention Peer Relationship Problems Prosocial Behaviour	7 years	Cord Blood: ↑ Pb ↓ Fluid IQ and ↓ Crystallized IQ (*β* =−0.12, 95% CI: −0.3; 0.02; and *β* =−0.14, 95% CI: −0.3; 0.007, respectively), and Mathematical skills (*β* =−0.15, 95% CI: −0.3; 0.01). Maternal Hair: On SDQ, ↑ Hg, ↑ Hyperactivity/Inattention problems (*β* = 0.18, 95% CI: 0.05 0.3) and ↑ Total difficulties (*β* = 0.14, 95% CI: 0.01; 0.3) No significant associations for Cd in cord blood or maternal hair
*Maternal urine*							
Dou et al. (2024)/ The Early Autism Risk Longitudinal Investigation (EARLI) and Markers of Autism Risk Learning Early Signs (MARBLES) [71]	USA	Mother–child pairs EARLI (*n* = 232), MARBLES (*n* = 425)	Sb, As, Ba, Be, Cd, Ce,Cr, Co, Cu, Pb, Mn, Hg, Mo, Ni, Pt, Se, Tl, W, U, V, Zn	1st/2nd trimester and 3rd trimester	Autism Diagnostic Observation Schedule DSM-5 diagnostic criteria Mullen Scales of Early Learning	3 years	1st/2nd trimester Cd concentrations > level of detection ↑ risk of ASD (RR = 1.69; 95% CI:1.08, 2.64); 1st/2nd trimester Cd concentrations > level of detection ↑ risk of non-typical development based on Mullen score one SD below average and ADOS scores within three point of the cutoff for diagnosis of ASD (RR = 1.29; 95% CI: 0.95, 1.75) No significant associations for As, Pb or Hg
Lozano et al. (2024)/INMA [72]	Spain	1003 mother–child pairs	MMA (monomethylarsonic acid), Cd, Co, Cu, Mo, Ni, Pb, Sb, Se, Tl, Zn	1st and 3rd trimester	Child Behavior Checklist (CBCL) Internalizing Problems Externalizing Problems Total Behavior Problems	9 years	↑ MMA (i.e. As) ↑ risk (i.e., IRRs; incidence ratio risk) of iInternalizing Problems (6.9%), Externalizing Problems (7.3%) and Total Behavior Problems (5.9%) ↑ Pb ↑ risk of Internalizing Problems (5.7%), Externalizing Problems (6.9%) and Total Behavior Problems (4.6%) In boys, ↑ Pb, ↑ Internalizing Problems , ↑ Externalizing Problems and ↑ Total Behaviour Problems (IRRs increase 7.3%, 9.6% and 5.1%, respectively) Associations between Pb and MMA and Total Behaviour Problems was modified by BDMF rs110301014 genotype
*Maternal blood and cord blood*							
de Assis Araujo et al. (2022) [12]	Brazil	48 mother–child pairs	As, Cd, Pb, Hg	3rd trimester maternal blood and cord blood at birth	Denver Developmental Screening Test-II (DDST-II)	- 6 months	Maternal Blood: ↑ As ↑ number of infants who failed DDST-II compared with infants that passed (Mann-Whitney *P*=0.03). Cord Blood: No significant associations for Cd, Pb and Hg
Kobayashi et al. (2022)/ Japan Environment and Children’s Study [73]	Japan	48,481 mother–child pairs	Hg, Se	maternal blood during pregnancy and cord blood at birth	Ages and Stages Questionnaire (ASQ)-3 Communication Gross Motor Function Fine Motor Function Problem Solving Personal–Social	0.5 to 4 years	Maternal Blood: Association between ↑ Hg ↓ problem-solving ability differed by parity Cord Blood: No association for Hg
*Maternal blood and urine*							
Nozadi et al. (2022)/ Navajo Birth Cohort Study (NBCS) [74]	USA	327 mother–child pairs	Whole blood: Pb, Cd, Hg, Mn Pb, Se Serum: Cu, Zn Urine: TAs, iAs (MMA, DMA), Ba, Be, Cd, Co, Ce, Hb, I, Mn, Pb, Mo, Pt, Sb, Sr, Sn, Tl, U, W	36 weeks GA or at birth	Ages and Stages Inventory Communication Gross Motor Function Fine Motor Function Problem Solving Personal–Social	10–13 months	↑ TAs ↓ Problem Solving (1 unit increase in TAs associated with -1.25 in score) ↑ Pb ↓ Fine Motor ( (1 unit increase in Pb associated with -0.90 in score) No significant associations for Cd or Hg
*Child blood*							
Wang et al. (2022) [70]	China	148 children	Pb, As, Cu	7.5 years	WISC-China Full Scale IQ (FSIQ) Verbal IQ (VIQ) Performance IQ (PIQ)	7.5 years	↑ Pb ↓ VIQ in girls (*β* = −0.491; 95% Ci: −0.863,–0.118) No significant associations for As
*Infant/ child fingernails/toenails*							
Bauer et al. 2024/ New Hampshire Birth Cohort Study (NHBCS) [30]	USA	268 children	As, Cu, Mn, Pb, Se, Zn	6 weeks of age	Behavior Assessment System for Children (BASC)-2	3 and 5 years	No significant associations for As and Pb
*Human milk*							
Naspolini et al. (2024)/ Germina cohort [75]	Brazil	185 mother–child pairs	As, Cd, Hg, Pb	3 months	BSID-III Cognitive Language Motor	3, 5–9, 10–16 months	↑ Pb ↓ Language development trajectory from 3 months to 10–16 months (*β* = -.0.413 95% CI: −0.653,–0.173) Infants exposed to Pb ↓ Language development at 10–16 months (*M* = 97.47, SD = 13.36) compared with non-exposed infants (*M* = 102.96, SD = 11.53). No significant associations for As, Cd or Pb
*Cord blood and infant toenails*							
Yang et al. (2022)/Shanghai Birth Cohort [33]	China	484 mother–child pairs	Hg, Se	Cord blood at birth and toenails within 6 months of birth	ASQ Communication Gross Motor Function Fine Motor Function Problem Solving Personal–Social	6–12 months	No significant associations for Hg only ↓ Hg ( < median 0.13 ppm) and ↑ Se ( ≥ median 0.87 ppm) in infants with ↑ long-chain omega-3 polyunsaturated fatty acids associated with ↑ Gross Motor Function
*Cord blood and child blood*							
Gagnon-Chauvin et al. (2023)/ Nunavik Child Development Study (NCDS) [76]	Canada	212 mother–child pairs	Hg, Pb and polychlorinated biphenyls (PCBs)	Cord blood at birth and child blood at 11 years and 18 years	Brief Sensation Seeking Scale (BSSS-4) Sensation Seeking– 2	18.5 years	Cord blood: No significant associations for Pb or H Child blood; BSSS-4 (lower global sensation seeking) ↑ Pb (b = -0.18, *P*=0.01) Adolescent blood: No significant associations for Pb or Hg
*Maternal blood, cord blood, child blood, child urine*							
Kampouri et al. (2024)/ Nutritional Impact on the Immunological Maturation during Childhood in relation to the Environment (NICE) cohort [77]	Sweden	470 mother– child pairs	Pb, Cd, fluoride	maternal blood at 29 weeks GA and cord blood	WPPSI-IV-Sweden CBCL Social Responsiveness Scale	4 years	No significant associations for Pb or Cd
*Meconium,* ** ** *child hair, child fingernails*							
Jiang et al. (2022) [32]	Taiwan	53 mother–child pairs	Cord blood and meconium at birth and child fingernale at 3 years	As, Cd and Hg	Bayley Scales of Infant Development (BSID)-III Cognitive Language Gross Motor Fine Motor	3 year	Meconium: No significant associations for As, Cd or Hg Child hair: ↑ As ↓ Gross Motor (β = −0.032, 95% CI; −0.061,–0.004) Child fingernails: No significant associations for As, Cd or Hg

Note: ASQ = Ages and Stages Questionnaire; As = Arsenic; Ba = Barium; BASC = Behavior Assessment System for Children; BSID = Bayley Scales of Infant and Toddler Development; Be = Beryllium; BSSS = Brief Sensation Seeking Scale; CBCL = Child Behavior Checklist; Cd = Cadmium; Ce = Cerium; CI = Confidence Interval; Cr = Chromium; Co = Cobalt; Cu = Copper; DDST = Denver Developmental Screening Test; FSIQ = Full Scale IQ; Hg = Mercury; LCn3PUFAs = Long Chain Omega 3 Polyunsaturated Fatty Acids; Mn = Manganese; MMA = Monomethylarsonic acid; MSEL = Mullen Scales of Early Learning; Pb = Lead; I = Iodine; Mo = Molybdenum; No = Nickel; PCB = polychlorinated biphenyls;;PIQ = Performance IQ;Pt = Platinum; Sb = Antimony, SDQ = Strengths and Difficulties Questionniare; Se = Selenium; Sn = Tin; Sr = Strontium; SE = Standard Error; Tl = Thallium; W = Tungsten; U = Uranium; V = Vanadium; VIQ = Verbal IQ; WISC = Wechsler Intelligence Scale for Children; WPPSI = Wechsler Preschool and Primary Scale of Intelligence; Zn = Zinc.

*Associations between environmental chemicals other than As, Cd, Pb and Hg, and child cognitive/general development, motor, behavioural and mental health outcomes can be found in Table S1.

#### Motor

Few studies have examined motor outcomes in relation to prenatal As exposure. One study reported that higher levels of maternal urinary As (i.e. > 9.5 ug/L) were associated with significantly poorer performance on a measure of motor proficiency in children aged 5.5 years [50]. A second study noted that high levels of As in child hair at three years were associated with poorer gross motor performance on the BSID-III at age three [32]

#### Behaviour and mental health

In a large Spanish cohort, higher maternal urinary concentrations of MMA, an aresenic species, were associated with increased risk of internalizing, externalizing and total behaviour problems [72] Dai et al. reported that higher levels of maternal urinary As were not associated with child behaviour problems at six years of age; however, sex-specific analyses revealed that higher urinary levels at six years were associated with more anxious/depressed problems in girls [66]. In contrast to these findings, Midya et al. reported that As levels in maternal blood were associated with lower levels of depressive symptom in children 9–11 years of age. Studies that included multiple examined and examined the individual associations with specific chemical reported no associations between prenatal or childhood exposure to As and behavioural outcomes (i.e. internalizing and externalizing behaviours, risk of autism spectrum disorder [ASD]) [30,71].

### Cadmium exposure and neurodevelopment

#### Cognitive/general development

A limited number of studies have investigated the associations between prenatal exposure to Cd and child cognitive and developmental outcomes. A single chemical study conducted using data from the Japan Environment and Children’s Study reported that in a sample of 96,165, mother–child pairs, maternal blood concentrations of Cd in the second or third trimester of pregnancy were associated with delayed child development on the Ages and Stages Questionnaire-Third Edition at six months and 1.5 years [38]. Delays in development were not seen at older ages (i.e. 2, 2.5 and 3 years) (Table 2). In a study that examined exposures to multiple chemicals, maternal urinary concentrations of Cd in the first/second trimester of pregnancy were associated with a 1.29 higher risk of non-typical development on the Mullen Scales of Early Learning in a population of children at risk for ASD (Table 3) [71]. In contrast, Kampouri et al. reported no associations between Cd concentrations in maternal blood and urine, cord blood or child blood and urine and child IQ at four years of age [77]. Furthermore, a number of studies that examined prenatal and child exposures to multiple chemicals in various biomarkers (i.e. maternal blood, cord blood, maternal hair, maternal urine, child hair, child fingernails) did not identify associations between Cd levels and child cognitive/general development outcomes [12,32,72,74,75].

#### Behaviour and mental health

One study reported that maternal blood concentrations of Cd in the first/second trimester of pregnancy were associated with a 1.69 higher risk of ASD, in a population at risk. However, Kampouri et al. reported no associations between Cd concentrations in maternal blood and urine, cord blood or child blood and urine and children’s behaviour outcomes in the CBCL or scores on the Social Communication Questionnaire (SSQ), a measure used to screen for ASD, at four years of age. Also, Midya et al. did not find an association between maternal Cd in the second or third trimester and childhood depression symptoms at 9–11 years (Table 3) [78]. Further, Gari et al. found no associations between maternal hair concentrations of Cd and children’s behaviour at seven years on the SDQ [31]

### Lead exposure and cognitive, behavioural and mental health outcomes

#### Cognitive/general development

Single chemical and multi-chemical studies investigating prenatal and/or childhood exposure to Pb in various biomarkers (i.e. maternal blood, cord blood, child blood) and children’s cognitive and general developmental outcomes consistently report adverse associations (see Tables 2 and 3) [40–42,46,55,57,59–61,67,70,75] For example, Jia et al. reported that higher maternal plasma concentrations of Pb in the first trimester of pregnancy were associated with a higher risk of cognitive delay at two years of age and these associations were stronger in girls [40]. Higher maternal concentrations of Pb in whole blood in early pregnancy have also been linked to lower receptive communication skills [42]. Wang et al. reported that cord blood levels of Pb were negatively associated with child PIQ at 7.5 years [76]. A study by Naspoli et al. reported that maternal milk concentrations of Pb at three months postpartum were associated with poorer language development in children to 16 months of age [75]. Min et al. found that higher Pb concentrations in child blood at four years was related to lower language skills at age 12, and that language skills fully mediated that association between Pb and substance use-related problems at 15 years of age [61]. In contrast, Kampouri et al. did not find any associations between maternal blood, cord blood or child blood levels of Pb and cognitive development at four years. Studies that examined prenatal and/or childhood exposures to multiple chemicals also did not report significant associations between Pb exposure and child cognitive/general development outcomes [12,74,76,77].

#### Motor

Higher levels of exposure to Pb in maternal blood or serum collected in the third trimester or at birth were associated with poorer fine motor skills at 10–13 months of age based on parent report [74].

#### Behaviour and mental health

Maternal and child exposures to Pb were also associated with behavioural and mental health outcomes. For example, Alampi reported that higher levels of Pb in maternal blood in the first trimester of pregnancy were associated with higher scores on the Social Responsiveness Questionnaire-2, which is indicative of a higher number and more severe behaviours associated with autism [39]. Reyes-Sanchez et al. reported a significant association between maternal patella Pb levels measured within 12 months of delivery and conduct and aggression problems in children 6–11 years of age [42], and Lorzano et al. found that higher levels of Pb in maternal urine collected in the first and third trimesters were associated with an increased risk of internalizing, externalizing and overall behaviour problems at nine years of age [72]. Also, higher Pb concentrations in cord blood or in child blood at four years of age were associated with poorer inhibitory control at 8-10 years [64]. Sears et al. reported that higher Pb in child blood at eight years was associated with more externalizing problems and poorer adaptive skills on the BASC-2 [62]. Higher Pb concentrations in child blood at 3-5 years have also been associated with child sleep problems at 11–13 years, with these associations being more evident in boys [58]. Finally, Min et al. reported that higher Pb concentrations in child blood at age four were associated with a greater likelihood of substance use at age 15 [61]. Interestingly, Gagnon-Chauvin et al. reported that higher levels of Pb in cord blood, child blood and adolescent blood were related to lower levels of sensation seeking in adolescents at 18.5 years of age [76]. In contrast, Kampouri et al. did not find any associations between maternal blood, cord blood or child blood levels of Pb and social communication at four years. Other studies that investigated prenatal and/or childhood exposures to multiple chemicals did not report significant associations between Pb exposure and child behavioural outcomes [30,68]

### Mercury exposure and child neurodevelopmental outcomes

#### Cognitive/general development

Research examining associations between prenatal and childhood mercury exposure and child cognitive outcomes reports inconsistent findings (see Tables 2 and 3). For example, a study conducted in the Seychelles reported that higher levels of cord blood MeHg were associated with lower MDI scores on the BSID at 20 months of age [48]. Thurston et al. also reported adverse associations between time weighted average exposure to MeHg in early childhood (six months to 5.5 years) measured in children’s hair and performance on measures of language and word retrieval at age 22 years [28]. In contrast, a study conducted in Canada found no associations between maternal first trimester blood Hg and child intelligence at three to four years; however, sex-specific analyses revealed that in girls only, higher cord blood Hg and child blood Hg at three to four years were associated with higher FSIQ on the WPPSI-III [43]. Kuraoka et al. reported that cord blood concentrations of MeHg and inorganic Hg were not associated with children’s developmental quotients on the Kyoto Scales of Psychological Development at ages two or four. Another study conducted in Japan also reported no associations between cord blood and child nail levels of MeHg and child intelligence at seven to eight years [47]. Similarly, another study, also conducted in Japan, reported no associations between concentrations of MeHg in cord blood or in child hair at seven to eight years and child intelligence at the same age [63]. Thurston et al. reported that MeHg was not associated with cognitive outcomes in an American cohort [29] Further, a study conducted in the Seychelles reported no significant associations between child measures of MeHg in hair and any measures of child neurodevelopment at age seven after adjusting for prenatal MeHg [65]. In several studies that included multiple metals, no associations were reported between prenatal and/or childhood concentrations of Hg or MeHg in various matrices (i.e. maternal blood, maternal hair, cord blood, child blood, child hair) and child cognitive outcomes [12,31,32,71,73,74,76,79] (see Table 3) .

#### Behaviour and mental health

Research that has examined prenatal and/or childhood exposure to Hg and/or MeHg suggests that they may be related to behavioural outcomes (see Table 3). In a study that investigated exposure to multiple metals, Gari et al. reported that higher Hg in maternal hair was associated with more hyperactivity and attention problems and more overall problems at seven years [31]. Consistent with this, Ehlinger et al. in a cohort study conducted in Mexico reported that higher levels of Hg in children’s hair at 6–12 years were associated with more severe ADHD-like symptoms; however, these associations differed by sex [26]. A study conducted in the Seychelles reported that higher levels of time weighted average exposure to MeHg in early childhood (six months to 5.5 years) were associated with poorer performance on the Continuous Performance Test, which assesses sustained attention, vigilance and impulsivity, at nine years [28] In contrast, Sarzo et al. reported no significant associations between total Hg measured in cord blood and children’s behavioural functioning (i.e. attention, internalizing problems and externalising problems) from early childhood to adolescence [49]. Similarly, in the MoBa cohort, no associations were found between maternal blood levels of MeHg and internalizing or externalizing behaviours in children at three and five years of age [44].

### Metal mixtures and cognitive, motor, behavioural and mental health outcomes

The findings of research exploring the associations between mixtures of chemicals that included As, Cd, Pb and Hg and child cognitive, motor, behavioural and mental health outcomes is mixed and differs depending upon the metals included in the mixtures and the statistical mixtures analyses employed (see Table 4). Various statistical approaches were used including: Bayesian kernel machine regression (BKMR), weighted quantile sums regression (WQS), quantile G computation (QGC), principal components analysis (PCA), latent profile analysis and structural equation modelling.

**Table 4:** Studies that examined joint associations between prenatal and/or childhood exposure to chemical mixtures including arsenic, cadmium, lead and/or mercury and cognitive, behavioural and mental health outcomes in children*

Study/ Cohort	Country	Sample size	Exposure	Time exposure measured	Outcome	Child age at outcome assessment	Main findings
*Maternal blood*							
Gu et al. (2024)/ Guangi Zhuang Birth Cohort (GZBC) [80]	China	211 mother–child pairs	As, Ba, Cd, Co, Cu, Cs, Fe, Mo, Mn, Ni, Pb, Rb, Sb, Sr, U, W, Zn	< 13 weeks GA	Wechsler Preschool and Primary Scale of Intelligence (WPPSI)-IV-China Full Scale IQ (FSIQ) Wechsler Intelligence Scale-Fourth Edition, (WISC)-V-China Full Scale IQ	-6 years	Bayesian kernel machine regression (BKMR): No significant associations Negative trend for non-essential metals In single chemical analyses, ↑ Pb and ↑ U associated with ↓ FSIQ. Weight quantile sums (WQS): ↑ mixture of 10 non-essential metals ↓ FSIQ scores (WQS: β = −8.463; 95% CI −14.449,–2.476); U, Sb and Pb major contributors to FSIQ Quantile g-computation (QGC): mixture of 10 non-essential metals FSIQ scores (β = −7.003; 95% CI: −12.928,–1.078); U, Sb, Rb, As, Pb, Cd and Cs FSIQ; Ba, W and Sr FSIQ.
Thilakaratne et al. / Project Viva (2023) [81]	USA	900 mother–child pairs	Neurotoxic metals: As, Ba, Cd, Cs, Hg, Pb, Sn, Sr Nutrients: Co, Cu, Mg, Mn, Mo, Se, Zn, B12 folate	1st trimester	3 years: Wide Range Assessment of Visual Motor Abilities (WRAVMA) – Visual Motor – Visual Spatial – Fine Motor Peabody Picture Vocabulary Test (PPVT)-III Wide Range Assessment of Memory and Learning (WRAML)-2 – Design Memoty – Picture Memory 8 years Kaufman Brief Intelligence Test (KBIT)-II – IQ – Verbal IQ (Crystalized) – Nonverbal IQ (Fluid) WRAVMA	3 years: 8 years:	QGC findings at 3 years: ↑ nutrient mixture (Co, Cu, Mg, Mn, Mo, Se, Zn, B12, folate) ↑ PPVT-III (receptive vocabulary) at 3 years (*β* = 1.58; 95% CI: 0.06, 3.10); association driven by Zn, Se and Mg. In females, ↑ nutrient mixture↑ PPVT-III scores at 3 years (β =;2.58, 95% CI: 0.39, 4.77); driven by Mg. QGC findings at 8 years: ↑ neurotoxic metal mixture ↓ Visual Motor scores at 8 years (β = −3.01; 95% CI: −5.55,–0.47); association driven by Ba and Cs. In males, ↑ neurotoxic metal mixture ↓ Visual Motor scores at 8 years (*β* = −3.98; 95% CI: −7.64,–0.33); driven by Ba.
Yu et al./ Women and Children’s Healthcare in Wuhan, Hubei Province, (2024) [82]	China	2887 mother–child pairs	Al, Co, Hg, Mo, Mn, Ni, Pb, Se, Sr, V	13 weeks	Bayley Scales of Infant and Toddler Development (BSID)-China Mental Development Index (MDI) Psychomotor Development Index (PDI)	2 years	WQS: ↑ chemical mixture ↑ cognitive delay (OR = 1.55; 95% CI: 1.23, 1.95); Pb, Mn, V main predictors accounting for 46.91%, 16.44% and 12.83% of the total weight Thyroid stimulating hormone (THS) mediated the associations between Mn (3.51%) and Pb (3.18%) and MDI THS mediated associations between V (4.99%), Mn (5.12%), and Pb (10.14%) and PDI.
*Cord blood/* *Cord serum*							
Fan et al. (2025)/ Complex Lipids in Mothers and Babies (CLIMB) cohort [83]	China	189 mother–child pairs	Al, As, Ca, Cd, Ce, Co, Cs, Cu Fe, Ga, Gd, La, Mn, Mo, Ni, Rb, Se, Sr, U. Y, Yb, Zn, Zr	At birth	BSID-China MDI PDI	1 year	WQS: ↑ metal mixture concentration ↓ MDI (β = −7.167; 95% CI −12.28,–2.06) and PDI (β = −5.972; 95% CI −10.27,–1.67) Se contributed most to↓ MDI (0.157), followed by Mn (0.140), Cu (0.124), and Zr (0.116). Mn contributed most ↓ PDI (0.170), followed by Zr (0.139), Cu (0.114), and Ca (0.098). BKMR: ↑ metal mixture ↓ MDI when the mixture was below the 45^th^ percentile compared with the median Conditional posterior inclusion probability (PIP) values for individual metals were low ranging from 0.019 to 0.207, suggesting that no one metal contributed to this negative effect overall effect of metal mixtures was not significant for PDI.
Oppenheimer et al. /New = Bedford cohort (NBC) (2022) [84]	USA	562 mother–child pairs	Metals: Mn, Pb, Organochlorides: dichlorodiphenyldichloroethylene (DDE), hexachlorobenzene (HBC), polychlorinated biphenyls (PCBs)	At birth	WRAML – 2	13–17 years	BKMR: ↑ chemical mixture ↓ verbal working memory
Rokoff et al./NBC (2022) [85]	USA	468 mother–child pairs	Metals: Mn , Pb Organochlorides: DDE, HBC, PBC	At birth	Connor Parent Rating Scale Oppositional Hyperactivitry Cognitive Problems/Inattention Anxious-Shy Perfectionism Social Problems Psychosomatic Symptoms Behavior Assessment System for Children (BASC)-2 Parent Report Teacher Report Self-Report of Personality (SRP)	8 years CPRS 15 years PRS TRS SRP	BKMR: 8 years ↑ chemical mixture ↑ Anxious-Shy score in females at 8 years ↑ chemical mixture ↓ psychosomatic symptoms at 8 years ↑ chemical mixture ↑ anxiety and depression scores at 15 years on BASC-2 SRP BKMR: 15 years: ↑ chemical mixture ↑ anxiety and depression scores on SRP ↑ chemical mixture ↓ somatic symptoms on SPR ↑ cord blood Pb ↑ SPR anxiety symptoms (*β* = 1.56; 95% CI: 0.50, 2.61). ↑ cord blood Mn ↑ SRP Depression in girls [e.g. 3.26 (95% CI: 0.27, 6.25) BASC-2 SRP
Rosolen et al. / Northern Adriatic Cohort II (NAC-II). (2025) [86]	Italy	607 mother–child pairs	Mn, Pb	At birth	BSID-III Cognitive Language Motor	18 months	Interaction effect between Mn and Pb associated with ↓ cognitive scores in boys (β = −5.78, 90% CI: −11.17; −0.40).
*Maternal urine*							
Kou et al. /ECLIPSES Cohort (2025) [87]	Spain	400 mother–child pairs	Cd, Hg, Ni, Pb	12th week GA	BSID-III Cognitive Language (Receptive, Expressive) Motor	40 days	BKMR: no associations WQS: ↑ metal mixture ↓ expressive language (β = −0.26, 95% CI: −0.44,–0.07); Cd and Ni identified as the most influential contributors
Qiu et al./ Jiangsu Birth Cohort (JBC) (2024) [88]	China	853 mother–child pairs	As, Ba, Cd, Ce, Co, Cr, Cs, Cu, Hg, La, Mo, Mn, Ni, Pb, Rb, Re, Sb, Se, Sn, Sr, Ti, Tl, V, U, Zn	8–14 weeks GA	BSID-III Screening Test	3 years	BKMR: ↑ metal mixture of V, Cu, Zn, Sb, Ce, and U ↑ risk of non-optimal gross motor development children with concentrations in the upper 90th percentile had 1.44 times (95 % CI: 1.04, 2.00) ↑ risk of non-optimal gross motor development as compared in children with concentrations at the 50th percentile
Rosa et al. 2024/ Programming of Intergenerational Stress Mechanisms (PRISM) , Boston cohort, First Thousand Days of Life (FTDL) cohort [89]	USA	326 mother– child pairs	As, Cd, Mg, Pb, Sb	During pregnancy	National Institutes Health Toolbox Cognitive Battery, Early Childhood (CECC)	3–11 years	No associations when data from the three cohorts were pooled H Bayesian WQS: ↑ metal mixture ↓ CECC scores in the PRISM New York City (*ß* = −3.67, 95% credible interval (CRI): −7.61,–0.01) and FTDL cohort ((*ß* = −3.76, 95% CRI: −7.66,–0.24); a trend was noted for the PRISM Boston cohort (*ß* = −3.24, 95% CRI: -6.77, 0.144); the largest contributors were As (36%) and Sb (19%).
Tsai et al. / Taiwan Maternal and Infant Cohort Study (TMICS) (2023) [90]	Taiwan	408 mother– child pairs	Metals: As, Cd, Co, Cu, Ni, Pb, Tl Phthlates: methyl phthalate (MMP), mono-ethyl phthalate (MEP), mono-*n*-butyl phthalate (MnBP), mono-isobutyl phthalate (MiBP), mono-2-ethylhexyl phthalate (MEHP), mono-2-ethyl-5-hydroxyhexyl phthalate (MEHHP), and mono-2-ethyl-5-oxohexyl phthalate (MEOHP)	3rd trimester	Child Behaviour Checklist (CBCL) (1/12–5) Emotionally Reactive Anxious/Depressed Somatic Complaints Withdrawn Attention Problems Aggressive Behavior Sleep Problems	4 years	QGC: ↑ metals and ↑ phthalates (*β* = 2.07; *P*=0.03; q = 0.05) ↑ anxious/depressed problems; the top three chemicals were Co, MEOHP and Pb ↑ metal and ↑ phthalates mixtures ↑ odds of autism spectrum problems in children; (OR = 2.68–3.11); Cd and Co were the top chemicals
Xie et al. / Wuhan Healthy Baby Cohort (2025) [91]	China	1088 mother–child pairs	Al, As, Cd, Cr, Ga, Mn, Ni, Pb, Rb, Tl, V	13.1 weeks GA	BSID –China MDI PDI	2 years	WQS: ↑ metal mixture ↓ MDI score (*β* = -3.47; 95% CI: −5.00,–1.95); Al contributed most to mixture effect (49.2%), followed by Ga (21.6%), Mn (11.8%), Cd (7.5%), and Cr (3.7%) WQS: ↑ metal mixture ↓ PDI score (*β* = -1.88; 95% CI: −3.32,–0.44); Al largest contributor (30.9%), followed by Pb (22.6%), As (19.3%), Cd (10.0%), Rb (9.1%). .
Yu et al. / Early Autism Risk Longitudinal Investigation (EARLI) and the Markers of Autism Risk in Babies Learning Early Signs (MARBLES)- (2024) [92]	USA	251 mother–child pairs EARLI = 154; Marbles = 97)	Pb, Hg, Mn, Se	1st, 2nd and 3rd trimester (pooled samples)	Social Responsivness Scale	3 years	BKMR: no significant associations
*Placenta*							
Tung et al. / Rhode Island Child Health Study (2022) [35]	USA	192 mother–child pairs	Cd, Co, Cu, Fe, Mn, Mo, Se, Zn	At birth	NICU Network Neurobehavioral Scale	24–72 hr	QGC: ↑ metal mixture ↑ odds of newborns being assigned to the atypical neurobehavioural profile (OR = 3.23; 95% CI = 0.92, 11.36); Cd had the largest effect
Zhou et al. /Ma'anshan Birth Cohort (2025) [36]	China	1586 mother–child pairs	As, Cd, Co, Hg, Pb, Se, Zn	At birth	WPPSI-IV_China FSIQ Verbal Comprehension Visual Spatial Fluid Reasoning Working Memory Procossing Speed	3–6 years	BKMR: ↑ metal mixture ↓ FSIQ and ↓ Fluid Reasoning ; Cd and As largest contributors ↑ metal mixture ↓FSIQ, ↓Visual Spatial and ↓ Fluid Reasoning in boys. QGC: No significant associations for FSIQ ↑ metals ↓ Visual Spatial (β, −1.195; 95 %CI = −2.343 to −0.047, *P*=0.041) and ↓ Fluid Reasoning (β, −0.990; 95 %CI = −2.075 to 0.095, *P*=0.074) in boys
Zhou et al./ Ma'anshan Birth Cohort (2025)	China	2154 mother–child pairs	As, Cd, Co, Hg, Pb, Se, Zn	At birth	Conners Abbreviated Symptom Questionnaire-China) The Clancy Autism Behavior Scale		BKMR: ↑metals mixture ↑ADHD symptom risk QGC: ↑ total metal mixture ↑ADHD symptoms (OR: 1.27, 95% CI: 0.97, 1.65); Cd largest contributor, followed by Cu, Co, Mn, Hg, As and Cr. No significant associations for metal mixtures and ASD
*Maternal blood and urine*							
Nyanza et al./Tanzania Mining and Health Cohort (2025) [93]	Tanzania	310 mother–child pairs	Whole blood: Hg, Cd, Pb Urine: As	2nd or 3rd trimester	Malawi Developmental Assessment Tool General development Language Gross motor Fine motor	4 years	QGC: mixture of ↑Pb, ↑Hg, ↑Cd and ↑As ↓ Gross motor by 17.78% (aPR = 0.822; 95% CI: 0.6994, 0.966), Language ability by 55.36% (aPR = 0.446; 95% CI: 0.313, 0.636) and General development by 13.36% (aPR = 0.866; 95% CI: 0.747,1.0
Puvvula et al./MIREC, HOME (2025) [94]	Canada, USA	617 mother–child pairs	44 chemical biomarkers (metals, organochlorine, organophosphates, polybrominated diphenyl ethers (PBDEs), polychlorinated biphenyls (PCBs), phenols, phthalates, PFAS, organophosphate esters	6–13th week GA (MIREC) 13–19^th^ wk GA (HOME)	WPPSI-III FSIQ	3 years (MIREC) 5 years (HOME)	QGC: not significant association BKMR: ↓ chemical mixture ↓ FSIQ trend association in males ↓ chemical mixture ↑ FSIQ trend association in females.
Yonkman et al. / MIREC (2023) [95]	Canada	517 mother– child pairs	Profiles: Metals: As, Cd, Hg, Mn, Pb Organochlorine pesticides (OCPs), β-HCH, DDE, Oxychloride, trans-noncchlor Organophosphate pesticide metabolites (OPPs): DEP, DETP, DMDTP, DMP, DMTP phthalate metabolites: MCPP, MEP, MnBP, MBzP, sum of DEHP metabolitesPoly chlorinated biphenyls (PCBs), PCB 118, PCB 138, PCB 153, PCB 170, PCB 180, PCB 187 Poly brominated diphenyl ether (PBDE): BDPE47, Cotinine MnBP, MBzP	6–13 weeks GA	WPPSI-III FSIQ	3 years	Latent profile analyses: No significant associations noted between profiles (*n* = 5) and FSIQ
*Child blood*							
Thilakaratne et al (2024) [96]	USA	349 -children	Co, Cu, Mg, Mn, Mo, Se, Zn, As, Ba, Cd, Cs, Hg, Pb, Sn, Sr	3 years	3 years: WRAVMA PPVT-III 8 years: WRAVMA WRAML -2 KBIT-2	3 Years; 8 Years	QGC: no significant associations
*Child urine*							
Liao et al./The Prediction of Allergy in Taiwanese Children (PATCH) (2024) [97]	Taiwan	220 children	As, Cd, Cr, Pb, Mn, V	≤1 year (*n* = 220). 5 years (*n* = 187)	CBCL Emotionally Reactive Anxious/Depressed Somatic Complaints Withdrawn Attention Problems Aggressive Behavior Sleep Problems	5 years	BKMR: ↑ metal mixture at ≤ 1 year ↑ anxious/depressed, withdrawal problems and sleep problems, at 5 years; urinary As main contributor
Notario-Barandiaran et al. /INMA cohort (2024) [11]	Spain	962 children	As species, Co, Cu, Mo, Pb, Se, Zn	4–5 years	McCarthy Scales of Children’s Abilities-Spanish (original scale) Global General Cognitive Global Verbal Global Perceptual-Performance Global Quantitivative Global Memory Global Motor McCarthy Scales of Children’s Abilities-Spanish (new scales) Executive functions (verbal, visual) Visual and verbal span Working memory Gross and fine motor function Cognitive function	4.0 to 6.4 years	Principal component analysis: PC1 mixture of ↑ Cu, ↑ Se, ↑ Pb, ↑ Zn associated with ↓ Global Verbal (*ß* = − 1.44; 95% CI: −2.76,–0.11), ↓ Executive functions (*ß* = − 1.46, 95% CI : − 2.72,–0.20), ↓ Verbal executive functions (*ß* = − 1.88, 95% CI: − 3.17,–0.59), and ↓ Working memory (*ß* = − 1.74, 95% CI: − 3.07,–0.40); observed association driven by Pb PCA 2: mixture of ↑ inorganic arsenic, ↑ monomethylarsonic acid, ↓ Global Motor (*ß* = − 0.94 95% CI: − 1.88, 0.00) and gross motor function (*ß* = − 1.41, 95% CI: − 2.36,–0.46). PC3: mixture of ↑ Mo, ↑ Co associated with ↑ Visual and verbal span (*ß* = 1.14, 95% CI = 0.16 to 2.12), and ↑ global motor (*ß* = 1.02, 95% CI: 0.03, 2.01) PC4: ↑ arsenobetaine ↑ Fine motor function (*ß* = 1.01, 95% CI = 0.11, 1.92)
*Maternal serum and child urine*							
Liu et al. Guangxi Birth Cohort (2022) [98]	China	703 mother–child pair	Al, As, Ba, Ca, Cd, Co, Cr, Cu, Fe, Mg, Mn, Mo, Ni, Pb, Rb, Sb, Se, Sn, Sr, Ti, V	Maternal serum: 1st, 2nd or 3rd trimester Child urine 2–3 years	Gesell Development Diagnosis Scale-China; Language Gross motor Fine motor Adaptive behaviour Social behaviour	2–3 years	BKMR: ↑ maternal serum Al, ↑ infant urinary Cd and ↑ urinary Se, ↓ Language Infant urinary Cd (PIP = 0.911) played a major role in language
*Maternal and child hair*							
Punamaki et al. (2025) [34]	Palestine	392 mother–child pairs	Al, As, Ba, Cd, Co, Cr, Cs, Hg, Mg, Mn, Pb, Sn, Sr, Ti, U, V, W	At birt	6 months: Minnesota Child Development Inventory 18 months: BSID-III-Arabic Cognitive Language Motor Social-Emotional	6–7 months (*n* = 392); 18 months (*n* = 386	Structural equation models: Three latent variables: carcinogen metals (As, Mo, Cr (VI), neurotoxic metals (Cd, Ce, Pb, Sn, Ti) and teratogen metals (Mn, U, W) ↑ carcinogen metals ↓ cognitive, ↓ sensorimotor and ↓ socioemotional development at 6 months (*ß* = −0.176, *t* = −2.266, - = 0.023) ↑ carcinogen metals (*ß* = −0.14, *t* = −2.926, - = 0.003) and ↑ teratogen metals ((*ß* = −0.12, *t* = −2.523, - = 0.12), ↓ cognitive, ↓ language, ↓ motor and ↓ socioemotional development at 18 months.

*Associations of individual metals with neurodevelopmental outcomes reported in Table S1.

Note: Al = Aluminum; As = Arsenic; Ba = Barium; BKMR = Bayesian kernel machine regression: BASC = Behaviour Assessment System for Children; BSID = Baylsy Scales of Infant and Toddler Development; Ca = Calcium; Cd = Cadmium; ,Ce = Cerium; CECC = National Institutes Health Toolbox Cognitive Battery, Early Childhood; CI = Confidence intervals; Co = Cobalt; Cr = Chromium; Cu = Copper; Cs = Cesium; Fe = iron; Ga = Gallium; Gd = Gadolinium; Hg = Mercury; KBIT = Kaufman Brief Intelligence Test ; La = Lanthanum; MDI = Mental Developmental Index; Mg = Magnesium, Mn = Manganese; Mo = Molybdenum; Ni = Nickel; Pb = Lead; PBC-152 = 2,2’,4,4’,5,5’-hexachlorobiphenyl; PDI - Psychomotor Development Index; IPIP = Posterior nclusion Probablity; QGC = Quantile G-computation; Rb = Rubidium; Re = Rhenium; Sb = Antimony; Se = Selenium; SE = Standard Error; Sn = Tin; Sr = strontium; Ti = Titanium; U = Uranium; V = Vanadium; W = Tungsten; WISC = Wechsler Intelligence Scale for Children; WPPSI =Wechsler Preschool and Primary Scale of Intelligence; WRAML = Wide Range Assessment of Memory and Learning ; WQS = Weight quantile sums; WRAVMA = Wide Range Assessment of Visual Motor Abilities ; Y = Yttrium; Yb = Ytterbium; Zn = Zinc; Zr = Zirconium.

#### Cognitive/general development

A study conducted in Tanzania by Nyanza et al. using QGC revealed that higher prenatal exposure to a mixture of As, Cd, Pb and Hg was associated with reduced general development and language outcomes in children aged three to four years. In a study using WQS, Yu et al. found that higher exposure to a metal mixture was associated with increased odds of cognitive delay and that Pb was one of the main predictors accounting for 46.91% of the total weight [82]. Fan et al. [83] used WQS to examine the mixture effect of 23 metals on children’s outcomes on the BSID at one year of age. They reported that higher exposure to the mixture had an overall negative effect on child neurodevelopmental outcomes and that Mn, zirconiium (Zr), calcium (Ca) and copper (Cu) and were the highest contributors to the MDI, while Se, Mn, Cu and Zr were the highest contributors to the PDI. Liu et al. reported that in a mixture that included 21 metals, BKMR revealed that maternal aluminum (Al) and infant Cd and Se were negatively associated with language development at two to three years, with child urinary Cd playing the most important role in this association [98]. A recent study examined the associations between first trimester exposure to metals and children’s intelligence at four years using BKMR, WQS and QGC [80]. In BKMR models, no associations were found between a metal mixture of all 17 metals (i.e. 7 essential, 10 non-essential) or the mixture of 10 non-essential metals and children’s intelligence [80]; however, higher exposure to non-essential metals was associated with a declining trend in child FSIQ; single exposure analysis revealed significant adverse effects for uranium (U) and Pb on child intelligence. In contrast, both WQS and QGC mixture models revealed that higher exposure to a mixture of 10 non-essential metals, which included As, Cd and Pb, was associated with significantly lower FSIQ scores. WQS identified Pb, U and antimony (Sb) as major contributors to lower child FSIQ, whereas QGC identified Pb, As and Cd along with U, Sb, rubidium (Rb) and Cs (cesium) as having an adverse effect on child IQ, whereas barium (Ba), tungsten (W) and strontium (Sr) were found to be positive contributors to FSIQ. In a study conducted in China, using placental samples, Zhou et al. found using BKMR that higher exposure to a metal mixture was associated with lower FSIQ and Fluid Reasoning with Cd and As being the biggest contributors to the mixture effect; sex-specific effects were noted for FSIQ, Fluid Reasoning and Visual Spatial reasoning with boys performing significantly poorer than girls [36]. Using QGC, Zhou did not find any significant associations for FSIQ; however, similar sex-specific effects were found for Fluid Reasoning and Visual Spatial reasoning in boys. Other studies using various mixture methods (i.e. BKMR, WQS, QGC, PCA, structural equation modelling) and biomarkers of exposure have reported associations between higher exposure to metal mixtures and poorer cognitive/developmental outcomes [11,34,35,84,86,87,91]. In contrast, two studies that utilized maternal blood to examine the influence metal mixture effects using QGC, BKMR and PCS did not report significant effects in relation to child FSIQ scores on the WPPSI-III [94,95]. It is notable that both studies included participants from the Canadian MIREC cohort; Puvvula et al. also included participants from the HOME cohort in the USA. Further, a study by Thilakaratoe et al. conducted in the USA did not find any associations between child blood metal mixtures and language and visual-motor outcomes at three years and visual-motor, memory and intelligence outcomes at eight years [96]

#### Motor

Using QGC, Nyanza et al. reported that prenatal exposure to a mixture of As, Cd, Pb and Hg was associated with poorer gross motor outcomes in children aged three to four years [93]. A recent study reported that higher exposure to a neurotoxic mixture that included As, Ba, Cd, Cs, Hg, Pb, Sn and Sr was associated with poorer visual-motor performance at eight years of age [81]. Qiu et al. reported that higher levels of a metal mixture that included vanadium (V), Cu, Zn, Sb, Ce and U, which was determined from maternal blood, was associated with an increased risk for non-optimal gross motor development at three years [88]

#### Behaviour and mental health

A study that investigated the mixture effect of child urinary concentrations of six metals (i.e. As, Cd, chromium (Cr), Pb, Mn V) at less than one year of age on behaviour at five years using BKMR revealed that As was the main contributor to internalizing behaviour problems, withdrawal problems and sleep problems measured using the CBCL [90]. Rokoff et al. found that higher exposure to a mixture of metals, organochlorides and polychlorinated biphenyls was associated with higher anxious-shy scores at eight years in females and higher anxiety and depression scores at 15 years [38]. A study conducted in Taiwan that used QGC reported that higher exposure to a mixture of metals and phthalates was associated with more anxious/depression problems on the CBCL, and that Co, MEOHP and Pb were the top three predictors [90]. This study also reported that higher exposure to this mixture of metals and phthalates was associated with higher likelihood of autism spectrum problems. In contrast, a study by Yu et al. did not find that a metal mixture of Pb, Hg, Mo and Se was associated with scores on the Social Responsivness Scale in a cohort of children at risk for autism [92].

## Discussion

We reviewed the findings of recent research conducted with prospective longitudinal epidemiological cohorts that examined the associations between prenatal and/or childhood exposures to As, Cd, Pb and Hg and child neurodevelopmental outcomes. Prospective cohort studies have several advantages including their ability to relate a specific outcome to a particular exposure, to establish temporal sequences, to follow change over time and provide stronger evidence for causal relationships [99]. Typically, they also have available multiple variables that can be used to control for potential confounders. The findings of the majority, but not all the studies included in this mini-review, suggest that prenatal and/or childhood exposure to As and Pb are detrimental to children’s cognitive, motor, behavioural and mental health outcomes. The findings that examined the neurodevelopmental effect of prenatal and/or childhood exposure to Cd, Hg and MeHg are less clear; however, they do provide some support for adverse associations between exposure to these metals and poorer neurodevelopmental outcomes.

Research examining prenatal exposure to As reported that even at low concentrations, exposure was associated with adverse neurodevelopmental outcomes, including lower intelligence, poorer executive function, poorer motor skills and a higher likelihood of behaviour problems in children and adolescents [11,51,52,54,93,97,100]. Further, the evidence suggests that these adverse effects are more strongly associated with exposure early in pregnancy, particularly in the first trimester [51,52,54]. Similarly, studies investigating associations between prenatal and/or childhood exposure to Pb and children’s cognitive and behavioural outcomes found that higher levels of exposure were associated with adverse cognitive outcomes, including lower intelligence and general development, and poorer episodic memory, working memory, spatial processing and mental flexibility [41,59,60]. Higher levels of exposure were also related to behaviour problems, including poorer inhibitory control, externalizing problems, conduct problems, greater substance use in adolescence and poorer sleep [27,39,56,58,64]. However, these associations differed by child sex [41,59,64] and time of exposure [27,64], and were influenced by factors such as maternal folate concentrations, microbial signatures and maternal stress during pregnancy [39,56,58,68].

Fewer studies investigated associations between prenatal and/or childhood exposure to Cd, and the results of these studies lack consistency. One study using data from the Japan Environmental and Children’s Study reported that prenatal exposure was associated with delayed development in gross motor and problem-solving skills at one to 1.5 years of age on the ASQ, a parent report that screens for developmental delay in children. However, delayed development was not seen at two years of age [38]. Dou et al. reported that Cd concentrations in early pregnancy were associated with a significantly higher risk of autism spectum disorder (ASD) and non-typical development in an at-risk population of younger siblings of children with ASD [71]. In a mixture analysis, Liu et al. found that infant urinary Cd was the highest contributor to adverse language outcomes in children two to three years of age [98]. In contrast, Kampouri et al. found no significant associations between prenatal and child exposure to Cd and child cognition, behaviour and social communication at four years [77]. Other studies have also reported no associations between prenatal and childhood exposures to Cd in various biological samples (whole blood, meconium, human milk, hair) and children’s cognition, hyperactivity and attention problems, depressive symptoms and motor skills [31,32,68,75].

Findings from recent research examining the associations between prenatal and/or childhood exposure to Hg and MeHg and child neurodevelopment are also inconsistent, with many studies reporting few associations. Kuraoka et al. using data from the Japan Environment and Children’s Study found no significant associations between methyl mercury (MeHg) and Hg concentrations in cord blood and children’s neurodevelopment at two and four years [47]. Similarly, Sarzo et al., using data from the Spanish INMA cohort, reported no association between THg exposure and children’s behavioural functioning assessed from early childhood to pre-adolescence [49]. They suggested that the nutrients in fish could offset the potential neurotoxic impact of Hg. Love et al. examined the contribution of both maternal and child genetic backgrounds to MeHg exposure and the association with neurodevelopment [48]. Their results suggested that a child’s ABC transporter genetics may influence prenatal MeHg exposure, and in turn neurodevelopment outcomes. In contrast, other studies have reported associations between higher levels of exposure to Hg and cognitive, executive function and attention problems [26,28,31].

### Mixture analysis

There is growing concern that exposure to metals mixtures during foetal life and early childhood, critical periods of development, could increase the risk of adverse neurodevelopmental outcomes. Biomonitoring studies indicate that humans are exposed to a range of diverse metals across the lifespan, often simultaneously. Examining the neurodevelopmental effects of early exposure to a single metal such as As, Cd, Pb or Hg could underestimate or overestimate the true impact due to potential interactions with other metals and chemicals, and that the analysis of metal and chemical mixtures provides more realistic information on the real-world effects of exposure to heavy metals such as As, Cd, Pb and Hg.

The present review included studies that used various mixture approaches, including BKMR, WQS, CGC, principal componenets analysis (PCA), latent profile analyses and structural equation modeling, to assess the associations between maternal and/or childhood exposure, via various biosamples (e.g., blood, urine, hair), t0 metal/environmental chemical mixtures and children’s cognitive, motor, behavioural and mental health. The selection of the various mixture approaches utilized was guided by the specific research question(s) of the study and the capability of each analytical mixture method to address the question(s) of interest. BKMR utilizes a non-parametric approach to model all interactions and association among the environmental chemicals examined and an outcome (e.g. intelligence). It includes both the non-linear and/or interactions in the exposure-outcome association. It allows for variable selection, which is an important feature when the number of environmental chemicals included is large and/or the environmental chemical exposures are highly correlated, which is common in child health research [101]. BKMR produces posterior inclusion probabilities (PIPs), which represent the probability that each chemical was selected for inclusion in the mixture based on a specific number of iterations [102]. Weighted Quantile Sum (WQS) allows the estimation of a mixture effect of numerous environmental chemicals associated with a specific outcome such as child intelligence [103]. This method converts chemical exposure values into quantiles so that they are on the same scale. A single index that combines these exposures is then created. This index is used in a regression model to estimate the overall effect of the chemical mixtures on outcomes. It also estimates the weights for each of the chemicals in the mixture, showing their contribution to the overall mixture effect [103]. This method assumes that the combined effects of these exposures are null or in the same direction and linear and additive; however, support for these assumptions is limited. Quantile G computation (QGC) attempted to address this by not assuming that all chemical exposure effects are in the same direction. It also allows for effects to be nonlinear and non-additive [104]. QGC focuses on examining the influence of the mixture of chemicals on outcomes such as intelligence rather than focusing on the individual chemicals. Both WQS and QGC assume linearity in their mixture models, whereas BKMR is highly flexible and able to model complex non-linear and non-additive associations [102,105]. Research examining metal mixtures has also used PCA, latent profile analyses and structural equation models. Principal components analysis reduces the dimensionality and identifies consistent patterns in datasets with a large number of chemicals. It allows for easier interpretation of chemical exposures on outcomes [106]. Latent profile analyses identified subgroups of metals that may be associated with different neurodevelopmental outcomes [95], while structural equations allow for the formation of latent or summary variables (i.e. constructs) and the investigation of their effects on child outcomes [34,107]. These different metal mixture analyses methods may result in findings that are not consistent due to the assumptions employed (i.e. non-linear and non-additive assumptions for BKMR versus linear and additive assumptions for WQS) and the specific metals included in the mixture analysis. The use of more than one statistical technique in a study, such as BKMR and QGC, can provide a more comprehensive and robust understanding of how environmental chemical mixture exposures influence child outcomes [80]. Further, consistent results across different methods support reliability and validity of the metal mixture effects [80].

It is notable that in most of the metal/chemical mixtures studies reported here, higher exposure to metal mixtures both prenatally and in childhood was associated with poorer cognitive/general development, motor delays and problems in behavioural and mental health (see Table 4). These findings provide strong support for the contention that higher exposure to non-essential metal mixtures that include As, Cd, Pb and Hg is associated with poorer outcomes. Further, in many of the mixture models, Cd, Pb and As were frequently noted to be strong contributors to adverse cognitive, motor, behavioural and mental health outcomes [11,36,37,80,85,87,89,91–93,97], whereas Hg was identified in fewer studies [36,37]. However, it is important to note that the metals identified as contributing the most to the association between the metal mixture and child outcomes are influenced by other metals and chemicals included in the mixture analysis [108].

There are a number of importance considerations with metal mixture analysis. First, the investigation of metal mixtures is associated with methodological challenges including high within-person variability of some chemicals, which could result in misclassification [108] . Second, is the high financial cost. Third, investigators must consider the biospecimen(s) (e.g. blood, urine, hair) and the sample population(s) [109], both of which could have an influence on cost and outcomes.

### Sex-specific effects

Sex-specific effects were reported in studies that examined prenatal and child exposure to As, Hg and Pb. For example, Dai et al. reported that higher levels of As in child blood at six years were associated with more anxious/depressed problems in girls at six years. Li found that higher levels of As in cord blood were associated with poorer working memory in boys [45] and Lv et al. reported that higher concentrations of As in third trimester maternal urine were associated with lower MDI scores on the BSID. For Pb, higher levels of in cord blood were associated with poorer inhibitory control in males at eight to nine years and higher childhood exposure at four years was associated with poorer inhibitory control in both males and females at nine years [64]. Higher levels of Pb in child blood have also been associated with poorer executive function, spatial processing and attention in males, but problems in episodic memory in females [110]. Finally, higher levels of child concentrations of Hg measured in child blood at three to four years of age were found to be associated with an increase in FSIQ in female children, but not male children [43]. It is possible that these sex-specific associations could be due to sex-specific placental and maternal physiological responses. Possible explanations are variations in placental hormone production and metabolism by foetal sex. Specifically, placentas of female fetuses are reported to produce higher levels of corticotropin-releasing hormone and may respond differently to the environmental exposures than those of male fetuses [111,112]. Further, many of the fundamental processes underlying neurotoxicity in heavy metals such as oxidative stress, dysregulation of gene exposure and epigenetic alterations are influenced by sex. Therefore, it is not unexpected that the influence of prenatal and/or child exposures to heavy metals would result in sex-specific effects [2]. Also, because of their endocrine-disrupting properties, toxic metals could have differing effects on brain development in males and females, and in turn, neurodevelopmental outcomes [113]. Male and female children may also be differentially exposed to and affected by environmental exposures, which may result in sex-specific differences in physiological processes, brain structure and function, and in turn neurodevelopmental outcomes [2,114].

### Potential mechanisms

During the prenatal period, the developing fetus is very sensitive to environmental exposures including exposure to non-essential metals such as As, Cd, Pb and Hg. These metals can pass the placental foetal-maternal barrier and enter foetal circulation, which allows them to cross the foetal blood-brain barrier [115,116]; however, research suggests that placental transfer of Cd is limited [116]. Toxic metals can accumulate in brain tissue and have adverse effects on the development of the foetal brain and nervous system, which in turn can influence later cognitive, motor and behavioural functioning and mental health [117]. In early childhood, infants and children are also exposed to As, Cd, Pb and Hg via various routes including breastmilk, food, water, inhalation and absorption through the skin, which can result in continued accumulations of metals in brain tissue during sensitive periods of development [118]. These exposures can disrupt neurotransmitter systems, induce oxidative stress, influence genetic susceptibility and cause epigenetic alterations in genes crucial for brain development [2,119], all of which can influence neurodevelopment and potentially result in poorer cognitive, motor, behavioural and mental health outcomes in children [120].

Toxic metals such as As, Cd, Pb and Hg can interfere with the synthesis, release and metabolism of neurotransmitters, leading to altered neuronal communication and impaired cognitive function. Pb interferes with NMDA receptor function, compromising synaptic plasticity. It also competes with calcium ions, which can disrupt neuronal excitability and synaptic transmission, and impair long-term potentiation, which is critical for memory formation [121]. Toxic metals can also induce oxidative stress, which has been associated with reduced foetal growth and neurological problems [7,122]. Associations between maternal urinary concentrations of As and elevated levels of the oxidative stress biomarker 8-isoprostaglandin F2α have been reported in pregnant women enrolled in the Navajo Birth Cohort study [123]. Hg targets thiol groups in proteins, inhibiting enzymes essential for antioxidant defenses and promoting oxidative stress. This could lead to neuronal apoptosis and synaptic dysfunction, which may contribute to cognitive deficits in children. Oxidative stress can also interfere with hormone production and signalling in the hypothalamic-pituitary-adrenal (HPA) axis. Disruption to the HPA axis during pregnancy has been linked to preterm birth, lower birth weight, altered foetal programming, poorer immune development, metabolic dysregulation, maternal mental health and long-term behavioural and emotional problems in offspring [124–127]. Excess oxidative stress can disrupt normal mitochondrial function [128] leading to decreased energy production [129]. As the main function of mitochondria is to provide energy to cells, disruption may affect the development of various organs and tissues in the developing fetus, including the brain. Research has reported associations between metal-induced mitochondrial dysfunction and neurodevelopmental disorders such as autism [130].

Children’s genetic background could also influence the associations between toxic metal exposures and neurodevelopmental outcomes. Previous research suggests that BDNF may be associated with some behaviour problems including ADHD and ASD [131,132]. Studies examining Hg have reported that different *BDNF* genotypes moderate the impact of early exposure to this metal on child neurodevelopment [133,134]. In research with the INMA cohort, associations between postnatal Hg exposure were observed for polymorphisms in the *GSTP1*, *BDNF* and *APOE* genes [135], and a recent study conducted in this cohort found that the associations between prenatal exposure to metals, including Pb and MMA (an arsenic species) and internalising problems were modified by BDNF genotype [72]. Exposure to As, Pb or Cd has also been associated with alterations in serum concentrations of BDNF [72,1376–138]. Finally, a recent study by Love et al. in the Seychelles Children Development Study Nutrition Cohort 2 demonstrated an association between polymorphisms ABC-transporters genes in children and prenatal exposure to MeHg, and that the association between cord MeHg and the MDI of the BSID differed significantly across the three genotypes of *ABCB1* rs10276499 [48]. These studies suggest that genetic factors could play an important role in influencing how exposure to heavy metals is related to neurodevelopmental outcomes.

Neurons undergo substantial epigenetic reprogramming during development, especially by covalent modification of DNA through methylation, and there is considerable evidence that environmental factors can influence this process [139,140]. There is a growing body of evidence that has linked exposure to As, Cd, Pb and Hg to epigenetic alterations including DNA methylation, histone modification and change in miRNA expression profiles [141,142]. These epigenetic alterations could be associated with changes in neurodevelopmental trajectories and may vary by sex, exposure levels and duration of exposure [143–145]. Lv et al. found that among children prenatally exposed to As, DNA methylation mediated the association between exposure and neurodevelopment [146]. Specifically, three cytidylyl phosphate guanosine positions (annotated to *ARMC5*, *KIAA1217* and intergenic region) were found to mediate this association. Sex-specific associations between prenatal exposure to Cd and adult cord blood DNA methylation levels have been reported [147]. In females, methylation changes were found in genes responsible for organ development and the bone morphology and mineralisation, whereas in males, these changes have been shown mainly in genes related to cell death due to apoptosis. Two studies using data from the Project Viva cohort and the ELEMENT cohort both reported the prenatal exposure to Pb was associated with lower cord blood methylation [120,148]. Further, the Seychelles Child Development Study found that prenatal exposure to MeHg was associated with epigenetic alterations in genes involved in the regulation of neurodevelopment, including BDNF, a glucocorticoid receptor (*NR3C1*) and a glutamate receptor subunit NR2B (*GRIN2B*). These studies suggest that exposure to As, Cd, Pb and Hg can induce changes in epigenetic mechanisms and that these changes could be linked to neuronal differentiation, synaptic plasticity, and ultimately cognitive, motor. behavioural and mental health outcomes in children and adolescents.

### Limitations of the cohort studies that examine metal exposures

We noted that exposure to heavy metals was evaluated in various biological samples across studies, including whole blood, plasma, serum, erythrocytes, cord blood, urine, meconium, placenta, nails, hair, bone and human milk, and some studies examined exposure in more than one biological sample. These different biological samples may reflect different types of exposure and exposure timeframes [149]. Urinary metals are frequently used as an indicator of recent exposure and they are considered as an appropriate biomarker for some metals, such as As and Cd [150]. However, for metals, such as Pb, concentrations in blood are considered more suitable for biomonitoring because they reflect the combination of exposure during the previous months and exposure over the past few years. For Hg, the biological sample that is tested (i.e. blood, urine, hair) depends on the form assessed (inorganic versus organic) and timeframe [151]. Studies included in this mini-review also differ in the measures used to assess cognitive, behavioural and mental health outcomes in children and the ages at which the children were tested. These methodological differences could contribute to the lack of consistency in results. Further, they challenge the generalizability of the findings beyond the cohorts in which the research was conducted. Future research is needed using standardized and validated protocols for exposure assessment to improve reliability. As child neurodevelopment is a constantly changing, studies need to use standardized assessment measures of cognition, motor skills, behaviour and mental health. This ensures that the outcomes are reliable and valid for children at the specific age that they are tested. Future research that follows children longitudinally from infancy through adolescence and into adulthood is needed to investigate the influence of prenatal and early childhood exposures to As, Cd, Pb and Hg on the trajectories of development of cognitive and motor skills, behaviour and mental health across the lifespan. Another limitation of the research literature to date is that most studies examining the influence of exposure to toxic metals on children’s development have been conducted in high-income studies. However, levels of exposure to toxic metals are higher in low and middle-income countries (LMICs) due to the limited resources and insufficient infrastructure available to regulate and minimize toxicant exposures. This could result in significantly higher levels of environmental exposure to toxic metals, which may be associated with more adverse developmental and behaviour outcomes in these populations of children [152]. Future research in LMICs is needed that investigates the associations between levels of exposure to As, Cd, Pb, Hg and other toxic metals in pregnant women and young children and children’s long-term cognitive, motor, behavioural and mental health outcomes.

## Conclusions

The review provides strong support for the contention that higher levels of prenatal and childhood exposure to As and Pb, and to a lesser extent Cd and Hg, and their mixtures are linked with adverse cognition, motor, behavioural and mental health in children. There is some suggestion that these effects may differ by child sex. These findings support the need for further prospective cohort studies with longitudinal follow-up into adulthood that examine both the individual and mixture effects of exposure to heavy metals. The influence of potential mitigating factors such as maternal and child nutrition on long-term outcomes is an area in need of future investigation. In addition, studies are needed that elaborate on the sexually dimorphic effects of prenatal and childhood exposure to toxic metals on children’s cognitive, motor, behavioural and mental health. Research clarifying potential biological mechanisms that underlie the link between metal exposures and child neurodevelopment, and the role that factors such as the microbiome and the psychosocial environment play in mediating and/or moderating these associations is needed to advance our understanding of the effects of heavy metal exposures on neurodevelopment in children and adolescents. These efforts could inform the development of interventions and public health strategies that could protect brain health and the cognitive, motor, behavioural and mental health outcomes of future generations worldwide.

**Summary Point**

- Prenatal and childhood exposure to As, Pb, Cd, Hg and MeHg could have lasting consequences on children's cognition, motor, behaviour and mental health.
- Higher levels of overall exposure to heavy metals mixtures are associated with poorer neurodevelopmental outcomes.
- The effects of exposure to heavy metals on neurodevelopment may differ by child sex.
- Biological mechanisms such as disruption of neurotransmitter systems, oxidative stress, genetic susceptibility and epigenetic alterations could account for the associations between exposure to heavy metals and child outcomes.
- Prospective epidemiological studies are needed to clarify the long-term effects of prenatal and childhood exposures to heavy metals on children’s neurodevelopment.

## Supplementary material

online supplementary table 1.

## Open Access

This article has been published open access under our Subscribe to Open programme, made possible through the support of our subscribing institutions, learn more here: https://portlandpress.com/pages/open_access_options_and_prices#conditional

## Abbreviations

ADHD: Attention Deficit/Hyperactivity Disorder
ASD: Autism Spectum Disorder
ASQ: Ages and Stages Questionnaire
ASQ:SE: Ages and Stages Questionnaire: Social Emotional
Al: Aluminum
As: Arsenic
AsB: Arsenobetaine
As-III: Arsenite
As-V: Arsenate
BASC: Behaviour Assessment System for Children
BASC: Behaviour Assessment System for Children
BKMR: Bayesian Kernel Machine Regression
BNT: Boston Naming Test
BOT-2: Bruininks-Oseretsky Test of Motor Proficiency- 2nd Edition
BSID: Bayley Scales of Infant and Toddler Development
BSSS-4: Brief Sensation Seeking Scale
Ba: Barium
Be: Beryllium
CBCL: Child Behavior Checklist
CCPT-3: Connors Continuous Performance Test–3rd Edition
CDD: Cognitive Developmental Delay
CI: Confidence Interval
CPRS-R: Connors Parent Rating Scale–Revised
Ca: Calcium
Cd: Cadmium
Ce: Cerium
Co: Cobalt
Cr: Chromium
Cs: Cesium
Cu: Copper
DDST-2: Denver Developmental Screening Test-II
DMA: Dimethyl arsenic acid
DMA: Dimethyl Arsenic Acid
FSIQ: Full Scale IQ
Fe: Iron
Ga: Gallium
Gd: Gadolinium
Hg: Mercury
I: Iodine
KBIT: Kaufman Brief Intelligence Test
LCn3PUFAs: Long Chain Omega 3 Polyunsaturated Fatty Acids
La: Lanthanum
MDI: Mental Development Index
MMA: Monomethyl arsenic acid
MSCA: McCarthy Scales of Children's Abilities
MeHg: Methyl Mercury
Mg: Magnesium
Mg: Magnesium
Mo: Molybdenum
Ni: Nickel
PBC-152: 2,2’,4,4’,5,5’-hexachlorobiphenyl
PDI: Psychomotor Development Index
PDI: Psychomotor Development Index
PIP: Posterior Inclusion Probablity
PPVT: Peabody Picture Vocabulary Test
Pb: Lead
QGC: Quantile G-Computation
Rb: Rubidium
Re: Rhenium
SDQ: Strength and Difficulties Questionnaire
SE: Standard error
SRS-2: Social Responsiveness Scale–2
Sb: Antimony
Se: Selenium
Se: Selenium
Sn: Tin
Sr: Strontium
Ti: Titanium
Tl: Thallium
U: Uranium
V: Vanadium
W: Tungsten
WISC: Wechsler Intelligence Scale for Children
WPPSI-IV: Wechsler Preschool and Primary Scale of Intelligence–Fourth Editi
WQS: Weighted Quantile Sums
WRAML: Wide Range Assessment of Memory and Learning
Y: Yttrium
Yb: Ytterbium
Zn: Zinc
Zr: Zirconium
aOR: Adjusted Odd Ratio
iAS: Inorganic Arsenic
iAS: inorganic Arsenic
sNDD: suspected Neurdevelopmental Delay
tAs: total Arsenic
