# Supplementary material for: Prenatal and childhood exposures to heavy metals and their associations with child cognition, motor skills, behaviour and mental health
Source: Essays Biochem. 2025 Dec 29;69(3):199–240. doi: 10.1042/EBC20253010 (PMC12794335; doi:10.1042/EBC20253010)
Supplement: online supplementary table 1. [file ebc-69-03-EBC20253010-s001.docx]

**Table S1.** Findings for metals other than As, Cd, Hg, MeHg and Pb in studies that examined associations between prenatal and/or childhood exposure to multiple chemicals and cognitive, motor, behavioural, and mental health outcomes in children.

| **Study/**  **Cohort** | **Country** | **Sample size** | **Exposure** | **Time exposure**  **measured** | **Outcome** | **Child age at outcome assessment** | **Findings for other metals** |
| --- | --- | --- | --- | --- | --- | --- | --- |
| *Maternal Blood* | | | | | | | |
| Farias et al. (2022)/ Mexico’s National Institute of Public Health [67] | Mexico | 200 mother-child pairs | Pb, Hg, Mn | 3^rd^ trimester | Bayley Scales of Infant and Toddler Development (BSID)-III   - Cognitive - Language - Motor | 1, 3, 6, and/or 12 mos. | - ↑ Pb ↓ Language (*β* = −0.15, 95% CI: −0.24–−0.56); association moderated by exposure to Hg and Mn |
| Midya et al (2024)/  PROGRESS [68] | Mexico | 112 mother- child pairs | Pb, As, Cd, Mn, Co, Zn,  Cr, Cs, Cu, Sb, Se | 2^nd^ and 3^rd^ trimesters | Child Depression Inventory-2 | 9 – 11 yrs | - Second trimester:  -↑ Zn ↑ depression (β = 0.04; 95% CI: 0.01, 0.06)   -↑ Cr ↓ depression (β = -0.03; 95% CI: -0.05, 0.00)   - Third trimester:   - ↑ Cr ↑ depression ((β = 0.03; 95% CI: 0.0, 0.05)  - ↑ Co ↓ depression (β = -0.04; 95% CI: -0.07 -0.02)  - ↑ Zn second trimester, ↓ Co third trimester, and ↑ abundance of two microbial taxa, Bacteroides fragilis and Faecalibacterium prausnitzii associated with ↑ depression (β = 0.14; 95% CI: 0.05, -0.23); CDI score 15.4% higher than other participants. |
| Reardon et al./ Alberta Pregnancy Outcomes and Nutrition (APrON) (2023) [69] | Canada | 424 mother-child pairs | Hg,  serum 25 perfluoroalkyl acids (PFAAs) | 2^nd^ trimester | BSID-III-Canada   - Cognitive - Language - Motor - Socio-Emotional - Adaptive Behavior | 2 yrs | - ↑ PFAAs perfluoroheptanoate (PFHpA) (β = −0.88, 95% CI: −1.7, −0.06) and ↑ perfluorododecanoate (PFDoA) (β = −2.0, 95% CI: −3.9, −0.01) ↓ Cognitive scores - ↑ PFHpA ↓ Socio-emotional scores (β = -0.85, 95 % CI: −1.7, −0.02). - ↑ PFOS (β = 2.8, 95 % CI: 0.17, 5.4 ) and ↑ PFHxS ↑ Adaptive Behavior scores (β = 2.2, 95 % CI: 0.63, 3.7). - Non-linear relationships between total PFOS (β = −4.4, 95% CI: −8.3, −0.43), and linear-PFOS (β = −4.0, 95% CI: −7.5, −0.57) and 1m-PFOS (β = −1.8, 95% CI: −3.3, −0.24) isomers ↓ Language scores. |
| *Cord blood* | | | | | | | |
| Wang et al (2022) [70] | China | 148 mother- children | Pb, Se, As, Cu, Mn, Cr | At birth | Wechsler Intelligence Scales for Children (WISC)-China:   - Full Scale IQ - Verbal IQ - Performance IQ | - 1. yrs | - ↑Cu ↓ FSIQ ((β=-0.031; 95% CI: -0.057, -0.005) - ↑ Cu ↓ PIQ (β=-0.031; 95% CI: -0.061, -0.001) - ↑ Se ↓ VIQ in boys (β=-0.147; 95% CI: -0.262, -0. 031) |
| *Cord Blood and Maternal Hair* | | | | | | | |
| Gari et al. 2022/ Polish Mother and Child Cohort (REPRO_PL). [31] | Poland | 436 maternal-child pairs | Pb, Cd, Hg, Se, Zn, Cu | At birth, 3^rd^ trimester | Intelligence and Development Scales (Poland)  Strengths and Difficulties Questionnaire (SDQ)   - Total Difficulties - Emotional Symptoms - Conduct Problems - Hyperactivity/Inattention - Peer Relationship Problems - Prosocial Behavior | 7 yrs | Cord Blood:   - ↑ Se ↓ Emotional symptoms (*β* =−0.13, 95% CI: −0.3; 0.004) - ↑ Zn ↓ Emotional symptoms ( *β* =−0.10, 95% CI: −0.2; 0.02) |
| *Maternal Urine* | | | | | | | |
| Dou et al. (2024)/ The Early Autism Risk Longitudinal Investigation (EARLI) and Markers of Autism Risk Learning Early Signs (MARBLES)  [71] | USA | Mothe -child pairs  EARLI (n = 232),  MARBLES (n = 425) | Sb, As, Ba, Be, Cd, Ce,  Cr, Co, Cu, Pb, Mn, Hg, Mo, Ni, Pt, Se, Tl, W, U, V, Zn | 1st/2^nd^ trimester and 3^rd^ trimester | -Autism Diagnostic Observation Schedule;  -DSM-5 diagnostic criteria  -Mullen Scales of Early Learning (MSEL) | 3 yrs | - 3^rd^ trimester: ↑ Ce ↑ risk of ASD (RR = 1.69 (95% CI: 0.97, 2.95) |
| Lozano et al. (2024)/  INMA [72] | Spain | 1003 mother- child pairs | As (monomethylarsonic acid; MMA), Cd, Co, Cu, Mo, Ni, Pb, Sb, Se, Tl, Zn | 1^st^ and 3^rd^ trimester | Child Behavior Checklist   - Internalizing Problems - Externalizing Problems - Total Behavior Problems | - yrs | - ↑ Cu ↑ risk of Internalizing, Externalizing and Total Behavior problems; IRRs (incidence ratio risk) of 4.6 to 7.5%. - ↑Co, ↑Mo, or ↑Ni, ↑ risk of Internalizing Problems (IRRs increase up to 8%) - ↑ Ni ↑ Internalizing Problems in boys (IRR increase 10.4%) - ↑ Se ↑ Total Behaviour Problems in girls (IRR increase 9.5%) |
| *Maternal blood and Cord blood* | | | | | | | |
| Kobayashi et al (2022)/ Japan Environment and Children’s Study [73] | Japan | 48,481 mother-child pairs | Hg, Se | Maternal blood during pregnancy and cord blood at birth | ASQ-3   - Communication - Gross Motor Function - Fine Motor Function - Problem Solving - Personal-Social | - 1. to 4 yrs | Maternal Blood   - ↑ Se ↓ Communication (OR = 2.65; 95% CI:1.30, 5.40), ↓ Gross Motor (OR = 2.51; 95% CI: 1.65, 3.82), ↓ Fine Motor (OR = 2.61; 95% CI: 1.68, 4.04), ↓ Problem Solving (OR = 2.39; 95% CI: 1.60, 3.64), and Personal- Social (OR = 2.08; 95% CI:1.18, 3.65) skills. - Association between ↑ Se and ↓ Communication, Personal- Social skills differed by child sex and parity (primiparous vs. multiparous).   Cord Blood   - ↑ Se ↓ Problem Solving (OR = 3.85; 95% CI: 1.01, 14.61). |
| *Maternal Blood and Urine* | | | | | | | |
| Nozadi et al. (2022)/ Navajo Birth Cohort Study (NBCS) [42] | USA | 327 mother-child pairs | -Whole blood: Pb, Cd, Hg, Mn  Pb, Se  -Serum: Cu, Zn  -Urine: TAs, iAs (MMA, DMA), Ba, Be, Cd, Co, Ce, Hb, I, Mn,  Pb, Mo, Pt, Sb, Sr, Sn, Tl, U, W | 36 wks GA or at birth | Ages and Stages Inventory (ASI) | - 1. os | - ↑ Cu ↓ Personal-social (-1.03). - ↑ Mo ↓ Communication   (-0.71)   - ↑ Sr ↑ Problem-Solving (1.26) - ↑ W ↑ Communication (0.76) |
| ***Child Blood*** |  |  |  |  |  |  |  |
| Wang et al (2022) [70] | China | 148 mother- children | Pb, As, Cu | 7.5 yrs | WISC-China:   - Full Scale IQ, - Verbal IQ - Performance IQ | 7.5 yrs | - -No significant associations for Cu |
| *Infant/ Child Fingernails/Toenails* | | | | | | | |
| Bauer et al 2024/ New Hampshire Birth Cohort Study (NHBCS)[30] | USA | 268 children | As, Cu, Mn, Pb, Se, and Zn | 6 weeks of age | BASC-2   - Total Behavior Problems - Externalizing Behaviors - Internalizing Behaviour | 3 to 5 yrs | - ↑ Cu ↓ Total Behaviour Problems (BSI: *β* = − 3.88; 95%CI: − 7.12, − 0.64), ↓ Externalizing Behaviours (*β* = − 2.20; 95%CI: − 4.07, − 0.33), and ↓ Internalizing Behaviours (*β* = − 1.24; 95%CI: − 3.24, 0.76)] from 3 to 5 yrs - ↑ Zn ↑ externalizing behaviours from 3 to 5 yrs (*β* = 3.42, 95%CI: 0.60, 6.25) |
| *Cord blood and child toenails* | | | | | | | |
| Yang et al (2022)/ Shanghai Birth Cohort [33] | China | 484 mother-child pairs | Hg, Se | Cord blood at birth and child toenails within 6 months of birth | ASQ:   - Communication - Gross Motor Function - Fine Motor, Function - Problem Solving - Personal-social skills | 6-12 mos: | - No significant associations for only Se |
| *Cord blood and child blood* | | | | | | | |
| Gagnon-Chauvin et al. (2023)/ Nunavik Child Development Study (NCDS) [76] | Canada | 212 maternal-child pairs | Hg, Pb and polychlorinated biphenyls (PCBs) | At birth; 11 yrs, 18 yrs | -Brief Sensation Seeking Scale (BSSS-4) Sensation Seeking– 2 | 18.5 yrs | Cord Blood   - ↑ PCB-153 ↓ BSSS-4 scores (b = -0.15, p = 0.04; b = -0.24, p = 0.004 (lower risk-related sensation seeking)   Child Blood   - ↑ PCB-153 (b = -0.16, p = 0.06) ↓ BSSS-4 (lower global sensation seeking)   Adolescent Blood   - ↑ PCB-153 ↓ SS2 lower risk of sensation seeking (b = -0.15, p = 0.04; b = -0.24, p = 0.004) |
| *Maternal blood, cord blood, child blood, child urine* | | | | | | | |
| Kampouri et al. (2024)/ Nutritional Impact on the Immunological Maturation during Childhood in relation to the Environment (NICE) cohort [77] | Sweden | 470 mother- child pairs | Pb, Cd, fluoride | 29 wks GA, at birth, maternal; blood, cord blood | -WPPSI-IV-Sweden;  -CBCL  - SSR-2 | 4 yrs | - No significant associations for fluoride |

Note: ASQ = Ages and Stages Questionnaire; As = Arsenic; Ba = Barium; BASC = Behavior Assessment System for Children; BSID = Bayley Scales of Infant and Toddler Development; Be = Beryllium; BSSS = Brief Sensation Seeking Scale; CBCL = Child Behavior Checklist; Cd = Cadmium; Ce = Cerium; CI = Confidence Intervals; Cr = Chromium; Co = Cobalt; Cu = Copper; DDST = Denver Developmental Screening Test; FSIQ = Full Scale IQ; Hg = Mercury; LCn3PUFAs = Long Chain Omega 3 Polyunsaturated Fatty Acids; Mn = Manganese; MMA = Monomethylarsonic acid; Pb = Lead; PBC-153 = PBC-152 = 2,2’,4,4’,5,5’-hexachlorobiphenyl; I = iodine; Mo = Molybdenum; No = Nickel; PIQ = Performance IQ; Pt = Platinum; Sb = Antimony, SDQ = Strengths and Difficulties Questionnaire; Se = Selenium; Sn = Tin; Sr = Strontium; SE = Standard Error; Tl = Thallium; W = Tungsten; U = Uranium; V = Vanadium; VIQ = Verbal IQ; WISC = Wechsler Intelligence Scale for Children; WPPSI = Wechsler Preschool and Primary Scale of Intelligence; Zn = zZinc.
